# Single-cell transcriptomic profiling of cerebrospinal fluid reveals immune-cell heterogeneity and multicompartment MIF/CD74-related communication in tuberculous and purulent meningitis

**DOI:** 10.3389/fcimb.2026.1867160

**Published:** 2026-08-13

**Authors:** Haoyang Hu, Lei Hou, Yan Wang, Zekai Yu, Jiatang Zhang, Fei Yang

**Affiliations:** 1 Department of Neurology, The First Medical Centre, Chinese People's Liberation Army General Hospital, Beijing, China; 2 Medical School of Chinese People's Liberation Army, Beijing, China; 3 School of Computer Science and Technology, Hangzhou Dianzi University, Hangzhou, Zhejiang, China

**Keywords:** CD4+ T cells, CD74, cerebrospinal fluid, MIF, purulent meningitis, single-cell RNA sequencing, tuberculous meningitis

## Abstract

**Background:**

Tuberculous meningitis (TBM) is a severe central nervous system infection in which dysregulated host inflammation contributes to neurological injury. The cellular organization of the cerebrospinal fluid (CSF) immune microenvironment in TBM, particularly in comparison with other inflammatory meningitis conditions, remains incompletely defined. This study characterized CSF immune-cell heterogeneity in TBM and purulent meningitis (PM) using single-cell transcriptomic profiling.

**Methods:**

Pre-treatment CSF immune cells from five patients with TBM and five patients with PM were profiled using 10x Genomics single-cell RNA sequencing. After quality control, ambient RNA assessment, and doublet filtering, 96,594 cells were retained. Major immune-cell populations and myeloid and granulocyte subclusters were annotated using canonical lineage markers and cluster-specific marker genes, with additional evaluation of doublet-related quality-control metrics and ambient RNA estimates. Immune-cell composition, CD4^+^ T-cell transcriptional states, macrophage migration inhibitory factor (MIF)/CD74 expression, CellChat-inferred communication, and in silico perturbation profiles were evaluated using donor-level comparisons where appropriate.

**Results:**

Seven major CSF immune-cell populations were identified, including CD4^+^ T cells, CD8^+^ T cells, myeloid cells, granulocytes, B cells, plasma cells, and natural killer T (NKT)-like cells. Immune-cell composition varied markedly across donors. Several PM donors exhibited myeloid- or granulocyte-rich profiles, whereas several TBM donors showed greater CD4^+^ T-cell representation; group-level differences were exploratory and did not consistently reach statistical significance. Reannotation of the innate immune-cell compartment identified broad macrophage-like, monocyte-like, neutrophil-like, and CD1C^+^ dendritic cell–like populations. Two originally defined myeloid subclusters displayed coordinated T-cell-associated transcriptional programs and were interpreted as potentially misclassified or technically mixed cells rather than bona fide myeloid states. CD4^+^ T cells occupied a continuous transcriptional landscape comprising central memory (CM)-like, cytotoxicity/senescence-associated, and activated effector memory (EM)-like states with overlapping transcriptional programs. MIF was broadly detectable across immune-cell compartments, whereas CD74 was most prominent in antigen-presentation-associated populations. CellChat identified MIF-related communication in both disease groups, supporting a shared multicompartment inflammatory communication program rather than a TBM-specific pathway. Exploratory in silico perturbation prioritized MIF- and CD74-associated transcriptional programs for future validation.

**Conclusions:**

This study provides a donor-aware single-cell transcriptomic atlas of the inflammatory CSF immune microenvironment in TBM and PM. The findings highlight substantial inter-individual heterogeneity, broad innate immune-cell patterns, overlapping CD4^+^ T-cell transcriptional states, and multicompartment MIF/CD74-related communication. These transcriptome-inferred findings are hypothesis-generating and require validation in larger cohorts using protein-level and functional approaches.

## 1 Introduction

Tuberculous meningitis (TBM), caused by invasion of the central nervous system (CNS) by *Mycobacterium tuberculosis* (Mtb), represents one of the most devastating forms of extrapulmonary tuberculosis (Wilkinson et al., 2017; Manyelo et al., 2021). Despite standardized anti-tuberculosis chemotherapy and adjunctive anti-inflammatory therapy, TBM continues to be associated with high mortality and substantial neurological disability (Thwaites et al., 2004; Marais et al., 2010; Wilkinson et al., 2017). Survivors frequently experience long-term neurological sequelae, including cognitive impairment, cranial nerve dysfunction, motor deficits, stroke-related disability, and other complications of CNS injury (Wilkinson et al., 2017; Rohlwink et al., 2019). These poor outcomes are driven not only by persistence of Mtb within the CNS, but also by dysregulated host inflammatory responses within the cerebrospinal fluid (CSF), meninges, cerebral vasculature, and adjacent brain compartments (Davis et al., 2019; Rohlwink et al., 2019; Davis and Wilkinson, 2021). A more detailed understanding of the cellular composition and transcriptional organization of the local CSF immune microenvironment is therefore essential for clarifying TBM immunopathogenesis, contextualizing TBM-associated immune features against broader inflammatory meningitis responses, and identifying rational directions for future host-directed therapeutic research (Cresswell et al., 2021; Davis and Wilkinson, 2021).

The CSF is a specialized immune compartment during CNS infection and neuroinflammation. It participates in immune-cell trafficking, antigen exposure, inflammatory signal exchange, and surveillance of meningeal and perivascular immune responses (Engelhardt and Coisne, 2011; Schafflick et al., 2020; Mapunda et al., 2022). Its immune ecology differs substantially from that of peripheral blood and pulmonary sites of tuberculosis infection because of the blood–brain barrier, blood–CSF barrier, restricted leukocyte entry, limited nutrient availability, and distinct local inflammatory and metabolic conditions (Engelhardt and Coisne, 2011; Schafflick et al., 2020; Ampie et al., 2022; Mapunda et al., 2022). CD4^+^ T-cell-mediated type 1 immune responses are central to host defense against Mtb infection, particularly through coordination of macrophage activation and antimycobacterial immunity (Sakai et al., 2014; Morgan et al., 2021). However, most current knowledge of anti-tuberculosis immunity has been derived from peripheral blood, lung tissue, lymphoid organs, or animal models (Sakai et al., 2014; Morgan et al., 2021; Chandra et al., 2022). By comparison, less is known about how immune cells, including CD4^+^ T cells, are transcriptionally organized within the specialized CSF microenvironment of patients with TBM.

Single-cell RNA sequencing has provided a high-resolution approach for dissecting immune-cell heterogeneity, cell-state diversity, and compartment-specific immune organization in CNS inflammatory diseases and infections (Heming et al., 2022; Xiao et al., 2022). Recent single-cell studies have begun to reveal immune remodeling in the CSF of patients with TBM and other CNS infections, including changes in lymphoid and myeloid compartments and altered inflammatory or effector-associated transcriptional programs (Farhadian et al., 2018; Straeten et al., 2022; Tram et al., 2025). However, despite these advances, several key issues remain insufficiently resolved. TBM-associated immune features need to be interpreted within the broader CSF immune-cell landscape rather than within isolated cell lineages, and donor-level heterogeneity is particularly relevant in small CSF single-cell cohorts, where pooled-cell analyses may overestimate disease-group differences. In addition, CD4^+^ T-cell states in inflammatory CSF are likely to represent overlapping transcriptional programs rather than discrete, functionally validated subsets. Importantly, the cell–cell communication networks that may coordinate these transcriptional states remain incompletely characterized.

Macrophage migration inhibitory factor (MIF) and CD74 are of particular interest in inflammatory immune regulation. MIF is a pleiotropic cytokine involved in both innate and adaptive immune responses, whereas CD74 participates in antigen presentation and can function as a receptor component for MIF-related signaling (Leng et al., 2003; Shi et al., 2006; Farr et al., 2020). Within this context, MIF/CD74-related signaling provides a biologically plausible framework for linking single-cell transcriptional states to multicellular inflammatory communication within the CSF immune microenvironment. However, in the setting of TBM, it remains unclear whether such signals represent TBM-specific mechanisms, are preferentially associated with CD4^+^ T cells, or instead reflect broader inflammatory communication programs shared across multiple CSF immune-cell compartments. Moreover, because ligand–receptor analyses based on single-cell transcriptomic data infer potential communication from gene-expression patterns rather than directly measuring protein-level signaling, these observations should be interpreted cautiously and should not be regarded as direct evidence of protein-level ligand–receptor binding, downstream pathway activation, or functional signaling interactions (Jin et al., 2021). Among the multiple signaling pathways inferred by CellChat, MIF-related communication was selected for focused, biologically motivated follow-up rather than because it was uniquely enriched in TBM or ranked highest by overall information flow. This pathway was prioritized because MIF was broadly expressed across lymphoid and myeloid compartments, whereas CD74 was concentrated in antigen-presentation-associated populations, providing a coherent candidate axis linking the adaptive and innate immune compartments highlighted by the present dataset.

In this study, we performed single-cell transcriptomic profiling of pre-treatment CSF immune cells from patients with TBM and purulent meningitis (PM). By using PM as an acute purulent inflammatory meningitis control condition, we aimed to contextualize TBM-associated immune features against shared inflammatory CSF responses rather than to identify TBM-specific biomarkers per se. We characterized the global CSF immune-cell landscape, examined innate and CD4^+^ T-cell heterogeneity, and explored MIF/CD74-related, transcriptome-inferred intercellular communication. Rather than defining experimentally validated functional subsets or disease-specific signaling mechanisms, this study provides a single-cell transcriptomic atlas of inflammatory CSF in TBM and PM and nominates candidate transcriptional and communication programs for future validation.

## 2 Methods

### 2.1 Study participants and CSF sample collection

This case–control single-cell transcriptomic study included pre-treatment CSF samples from five patients with TBM and five patients with PM. All patients were hospitalized in the Department of Neurology, the First Medical Center of the Chinese PLA General Hospital. TBM was diagnosed according to the international uniform case definition for tuberculous meningitis, in combination with compatible clinical manifestations, CSF biochemical profiles, neuroimaging findings when available, and microbiological or molecular evidence of Mycobacterium tuberculosis. PM was diagnosed based on clinical features consistent with acute PM, inflammatory CSF findings, and microbiological or molecular evidence of a bacterial pathogen. All included patients had pathogen-detection results compatible with the final clinical diagnosis.

To minimize treatment-related alterations in the CSF immune landscape, CSF samples were collected during diagnostic lumbar puncture before the administration of antimicrobial agents, anti-tuberculosis therapy, glucocorticoids, immunosuppressive agents, or other disease-modifying treatments. Patients were excluded if they had HIV infection, other central nervous system infections, malignant tumors, autoimmune diseases, systemic disorders that could substantially affect immune status, prior exposure to glucocorticoids or immunosuppressive agents before CSF sampling, marked blood contamination of CSF, or insufficient cell viability for single-cell RNA sequencing.

The study was conducted in accordance with the Declaration of Helsinki and was approved by the Ethics Committee of the First Medical Center of the Chinese PLA General Hospital (approval number: S2024-326-01). Written informed consent was obtained from all patients or their legally authorized representatives. Clinical characteristics, pathogen-detection results, treatment status before CSF sampling, comorbidities, CSF laboratory parameters, and cytokine measurements are summarized in **Supplementary Tables 1**–**S3**.

### 2.2 Single-cell suspension preparation, library construction, and sequencing

Fresh CSF samples were transferred to the laboratory at 4 °C immediately after collection, and single-cell suspension preparation was initiated within 1 hour after lumbar puncture. Cells were enriched by low-speed centrifugation at 400 × g for 5 min and gently resuspended in prechilled phosphate-buffered saline supplemented with 0.04% nuclease-free bovine serum albumin. Cell viability was assessed using trypan blue exclusion before library construction. Only samples with cell viability greater than 85% were processed for downstream single-cell RNA sequencing.

Single-cell suspensions that passed quality assessment were loaded onto the 10x Genomics Chromium Next GEM platform using the Chromium Next GEM Single Cell 3′ Gene Expression v3.1 chemistry with dual indexing. Droplet generation, reverse transcription, cDNA amplification, and library construction were performed according to the manufacturer’s protocol. The resulting libraries were sequenced on an Illumina NovaSeq 6000 platform using paired-end sequencing. Per-sample raw Cell Ranger cell calls and post-quality-control cell numbers are summarized in **Supplementary Table 4**.

### 2.3 Primary data processing and quality control

Raw FASTQ files were processed using Cell Ranger software (v8.0.1; 10x Genomics). Sequencing reads were aligned to the human GRCh38 reference genome, and gene–cell count matrices were generated separately for each sample. Downstream analyses were performed in R (v4.2.2) using Seurat (v4.2.2), Harmony (v0.1.1), SoupX (v1.6.2), DecontX implemented in celda (v1.16.1), scDblFinder (v1.12.0), DoubletFinder (v2.0.3), CellChat (v1.6.1), DESeq2 (v1.38.3), clusterProfiler (v4.6.2), and scTenifoldKnk (v1.0.3).

Quality control was performed independently for each sample before data integration. Cells were retained for downstream analysis if they contained 200–5,000 detected genes and had a mitochondrial transcript percentage below 10%. Cells with extreme Unique Molecular Identifier (UMI) count outliers were further inspected on a sample-by-sample basis and excluded when abnormally high UMI counts were accompanied by transcriptional features suggestive of multiples. To reduce technical artifacts associated with droplet-based single-cell sequencing, ambient RNA contamination and doublet events were assessed before sample integration.

Ambient RNA correction was performed using SoupX, with sample-specific ambient RNA profiles estimated from the raw and filtered feature-barcode matrices. DecontX was additionally used to estimate cell-level contamination fractions and to assess the robustness of the ambient RNA evaluation.

Doublet detection was performed independently for each sample, with scDblFinder used as the primary method and DoubletFinder applied as a sensitivity analysis. High-confidence predicted doublets were excluded before sample integration. Per-sample raw Cell Ranger cell calls and final post-quality-control singlet counts are reported in **Supplementary Table 4**. Because the archived workflow output did not retain stepwise counts that separately distinguished scDblFinder-predicted doublets from cells removed by other quality-control criteria, the difference between the raw and final cell counts was not interpreted as a doublet-specific removal count. Per-donor scDblFinder score distributions and the post-filtering classification of retained cells are shown in **Supplementary Figure 1**.

After quality control, ambient RNA assessment, and singlet filtering, 96,594 cells were retained for downstream analyses. The distributions of nFeature_RNA, nCount_RNA, percent.mt, and scDblFinder doublet scores were examined across donors. The post-filtering UMAP was additionally colored by doublet classification to confirm that cells retained in the final analysis object were classified as singlets.

### 2.4 Normalization, integration, dimensionality reduction, and clustering

Post-quality-control gene–cell count matrices were analyzed using the Seurat framework. Single-cell expression data were normalized and variance stabilized using SCTransform. Highly variable genes were identified, and principal component analysis was performed on the normalized expression matrix. Unless otherwise specified, the first 30 principal components were used for downstream integration, shared nearest-neighbor graph construction, clustering, and uniform manifold approximation and projection (UMAP) visualization.

To reduce donor-associated batch effects while preserving major biological structure, Harmony integration was performed using donor identity as the batch variable. Harmony-corrected embeddings were subsequently used for shared nearest-neighbor graph construction, unsupervised clustering, and downstream UMAP visualization.

To assess whether the global transcriptional structure before integration was visibly associated with technical quality-control covariates, the pre-Harmony UMAP embedding was colored by the number of detected genes per cell (nFeature_RNA), unique molecular identifier counts per cell (nCount_RNA), and mitochondrial transcript percentage (percent.mt). Harmony integration performance was evaluated separately using UMAP visualizations colored by donor identity and disease group before and after correction.

Integration quality was further assessed using the local inverse Simpson’s index (LISI), including cell-type LISI (cLISI) and integration LISI (iLISI). cLISI was used to assess preservation of biological cell-type structure, whereas iLISI was used to evaluate mixing of cells from different donors after Harmony correction. These quality-control and integration assessments are shown in **Supplementary Figure 1**.

### 2.5 Major immune-cell annotation and donor-level composition analysis

Major CSF immune-cell populations were annotated using an integrated strategy that combined unsupervised clustering, canonical marker-gene expression, and cluster-specific differentially expressed genes. Seven major immune-cell populations were identified: CD4^+^ T cells, CD8^+^ T cells, myeloid cells, granulocytes, B cells, plasma cells, and NKT-like cells.

CD4^+^ T cells were annotated based on the expression of CD3D, CD3E, CD4, and IL7R, whereas CD8^+^ T cells were annotated based on CD3D, CD3E, CD8A, and CD8B. Myeloid cells were defined by the expression of LYZ, CD14, CD68, and CST3, and granulocytes by CSF3R, FCGR3B, S100A8, and S100A9. B cells were annotated based on CD79A and MS4A1, whereas plasma cells were identified by MZB1, JCHAIN, and immunoglobulin-related genes. NKT-like cells were annotated as cells showing combined T-cell marker expression and natural killer cell-associated transcriptional features, including NKG7, GNLY, KLRD1, and cytotoxic effector genes, without a clear conventional CD4^+^ or CD8^+^ T-cell transcriptional identity. This annotation was therefore used to describe an NKT-like transcriptional phenotype rather than to define a functionally validated NKT-cell population.

For donor-level immune-cell composition analysis, cell counts and proportions were calculated for each major immune-cell population within each donor. Proportions were calculated relative to the total number of annotated major immune cells in each donor. Major immune-cell populations not detected in a given donor were assigned a value of zero for donor-level proportion comparisons. Donor-level major immune-cell counts and proportions are provided in **Supplementary Table 5**.

### 2.6 Subclustering of myeloid cells, granulocytes, and CD4^+^ T cells

To further characterize innate immune-cell heterogeneity within the cerebrospinal fluid microenvironment, the initially annotated myeloid and granulocyte compartments were extracted from the integrated dataset and subjected to secondary clustering separately. The original unsupervised myeloid and granulocyte subcluster assignments were retained as the primary computational output for subcluster-level marker-gene and donor-composition analyses.

To provide biologically interpretable immune-cell identities, the combined innate immune-cell compartment was subsequently re-evaluated using cluster-specific differentially expressed genes and coherent lineage-associated marker programs. Broad macrophage-like, monocyte-like, neutrophil-like, and CD1C^+^ dendritic cell–like identities were supported by the expression of C1QA/C1QB/CD68, CD14/LYZ, CSF3R/FCGR3B, and CD1C/FCER1A, respectively. Canonical marker FeaturePlots, representative marker-gene dot plots, and donor-level subcluster distributions are shown in **Supplementary Figure 2**. Available marker-gene tables for the original myeloid and granulocyte subclusters, including average log_2_ fold changes, raw and adjusted P values, and the percentages of cells expressing each gene within and outside the corresponding subcluster, are provided in **Supplementary Data S1**.

Marker-gene profiling showed that two originally defined myeloid subclusters, Myeloid_2 and Myeloid_5, expressed coordinated T-cell-associated transcriptional programs rather than isolated low-level TRAC expression. Myeloid_2 expressed T-cell-associated genes including IL7R, GZMK, LTB, TRAC, and CD3D, whereas Myeloid_5 expressed GZMA, CD3D, CD3E, TRAC, CCL5, GZMK, CD2, and IL32. These subclusters were therefore further evaluated using broader lymphoid- and myeloid-lineage marker profiles, scDblFinder scores, nFeature_RNA, nCount_RNA, post-filtering doublet classifications, and ambient RNA contamination estimates. The coordinated expression of multiple T-cell-lineage genes was considered compatible with lymphocyte misclassification, lymphocyte contamination, or residual mixed droplets, although a contribution from ambient RNA could not be excluded. Because the available transcriptomic and quality-control data could not definitively distinguish among these possibilities, these subclusters were not interpreted as bona fide myeloid states or as evidence of a novel TRAC-positive myeloid population.

Exploratory differential-expression analyses comparing tuberculous meningitis and purulent meningitis were performed separately within the myeloid and granulocyte compartments. Given the limited number of donors, substantial donor-level heterogeneity, and the non-independence of cells derived from the same donor, cell-level differential-expression results were used primarily for exploratory visualization and hypothesis generation and were not interpreted as definitive disease-specific transcriptional signatures.

CD4^+^ T cells were extracted from the integrated dataset and reclustered to characterize transcriptional heterogeneity within the inflammatory cerebrospinal fluid microenvironment. Unsupervised reclustering initially identified six clusters, designated CD4_C0 to CD4_C5, which were retained as the primary computational output. Comparison of cluster-specific marker-gene profiles showed that several clusters shared related core marker genes and transcriptional programs and occupied adjacent or continuous regions of the uniform manifold approximation and projection embedding without clear transcriptional boundaries.

Clusters showing concordant expression of state-associated marker genes and broadly similar module-score patterns were therefore consolidated into three broader transcriptional states to facilitate biological interpretation: a central memory (CM)-like CD4^+^ T-cell state, a cytotoxicity/senescence-associated CD4^+^ T-cell state, and an activated effector memory (EM)-like CD4^+^ T-cell state. This consolidation was not based on a single prespecified numerical threshold or a supervised classifier but represented a biologically guided descriptive summarization supported primarily by shared marker-gene expression, together with embedding continuity and concordant transcriptional programs. The broader labels were intended to describe related regions of a continuous transcriptional landscape rather than functionally validated or mutually exclusive CD4^+^ T-cell subsets.

The term “cytotoxicity/senescence-associated” was used because cytotoxicity- and senescence-associated transcriptional programs partially overlapped within this landscape. Similarly, “activated EM-like” denotes an activated effector memory–like transcriptional pattern rather than a canonical effector memory subset defined by surface-marker expression or functional assays. Additional visualizations colored by donor identity, disease group, unsupervised cluster assignment, and final transcriptional-state annotation were generated to assess whether the observed CD4^+^ T-cell structure was primarily driven by individual donors or disease-group separation. Canonical cytotoxicity-associated genes and exhaustion- and senescence-associated genes were also examined across the three annotated CD4^+^ T-cell states.

### 2.7 Module-score analysis

Module-score analysis was performed to evaluate activation-, cytotoxicity-, exhaustion-, senescence-, interferon-stimulated gene (ISG)-, and Treg-associated transcriptional programs across annotated CD4^+^ T-cell states. Scores were calculated at the single-cell level using the AddModuleScore function in Seurat with default control-gene binning. Gene sets were curated based on canonical immunological markers and prior literature.

The activation-associated gene set included CD69, CD38, HLA-DRA, HLA-DRB1, IL2RA, TNFRSF9, and ICOS. The cytotoxicity-associated gene set included GZMA, GZMB, GZMK, PRF1, GNLY, NKG7, CTSW, KLRK1, CCL5, and CST7. The exhaustion-associated gene set included PDCD1, LAG3, HAVCR2, TIGIT, TOX, CTLA4, ENTPD1, and CXCL13. The senescence-associated gene set included KLRG1, DPP4, A2M, B3GAT1, GZMK, and CX3CR1. The ISG-associated gene set included ISG15, IFITM1, IFI44L, IFIT1, IFIT3, MX1, OAS1, OAS2, and STAT1. The Treg-associated gene set included FOXP3, IL2RA, IKZF2, CTLA4, TIGIT, TNFRSF18, and CCR8.

To reduce pseudoreplication from treating individual cells as independent biological replicates, single-cell module scores were summarized as donor-level mean scores within each annotated CD4^+^ T-cell state. Donor-level module scores were then compared between TBM and PM groups. These analyses were used to support conservative transcriptional-state annotation and to characterize broad transcriptional programs within the CD4^+^ T-cell compartment. They were not intended to infer experimentally validated cytotoxic, exhausted, senescent, or regulatory T-cell function.

### 2.8 MIF/CD74 expression analysis and assessment of CD74-detectable CD4^+^ T cells

MIF and CD74 expression were examined across major CSF immune-cell populations and annotated CD4^+^ T-cell transcriptional states using dot plots, violin plots, and donor-level summaries where appropriate. This global expression analysis was performed to determine whether MIF and CD74 signals were restricted to the CD4^+^ T-cell compartment or distributed across multiple immune-cell populations within the inflammatory CSF microenvironment.

Because CD74 is closely associated with antigen-presenting cell biology, detectable CD74 expression within CD4^+^ T-cell states were interpreted cautiously. CD74-detectable cells were defined as cells with detectable CD74 transcripts in the normalized expression matrix. To assess whether CD74 signals in CD4^+^ T cells could reflect contamination from antigen-presenting cells, ambient RNA, or doublet events, CD74-detectable and CD74-undetectable cells within the cytotoxicity/senescence-associated CD4^+^ T-cell state were compared with respect to canonical T-cell markers, cytotoxicity-associated markers, B-cell markers, plasma-cell markers, myeloid-cell markers, granulocyte markers, predicted doublet scores, and ambient RNA contamination estimates.

### 2.9 Cell–cell communication analysis

Cell–cell communication was inferred using CellChat. Analyses were performed at the global immune-cell level and, where appropriate, within the CD4^+^ T-cell compartment. TBM and PM samples were analyzed separately and then compared to assess whether inferred communication patterns were shared across inflammatory CSF microenvironments or preferentially associated with one disease group.

For each analysis, normalized expression values from the non-integrated RNA assay were used as input, together with the annotated cell identities. Harmony-corrected embeddings were used for clustering and visualization but were not used as the expression input for ligand–receptor inference. Putative ligand–receptor interactions were inferred using the CellChat human ligand–receptor database. Cell groups containing fewer than 10 cells were excluded from communication inference. Communication probability, pathway-level signaling activity, and information flow were calculated according to the standard CellChat workflow.

MIF-related communication was evaluated at both the pathway level and the ligand–receptor-pair level, with particular attention to MIF–CD74 interactions across sender–receiver cell-type pairs. Global immune-cell-level analyses were used to evaluate the multicellular distribution of MIF-related communication across the inflammatory CSF immune landscape.

### 2.10 Exploratory in silico perturbation analysis

Exploratory in silico perturbation analysis was performed using scTenifoldKnk to evaluate potential transcriptional dependencies associated with MIF and CD74. Based on the observed expression patterns and CellChat-inferred communication results, virtual perturbation of MIF was performed in CD4^+^ T cells, whereas virtual perturbation of CD74 was performed in myeloid cells. These compartments were selected because they represented major MIF- or CD74-expressing immune compartments in the present dataset and because the analysis was designed to generate mechanistic hypotheses rather than to define definitive cell-type-specific regulatory mechanisms.

For each selected compartment, normalized single-cell expression matrices were used to reconstruct gene regulatory networks. Computational perturbation was then performed by removing the target gene from the inferred network and estimating the resulting regulatory-distance changes among connected genes. Genes were ranked according to predicted regulatory-distance changes after virtual perturbation. Gene Ontology enrichment analysis was performed on perturbation-associated gene sets using clusterProfiler, with Benjamini–Hochberg adjustment for multiple testing. Predicted regulatory-distance rankings, enriched biological processes, and perturbation-associated regulatory networks were generated to prioritize candidate transcriptional programs potentially linked to MIF or CD74. Because scTenifoldKnk infers perturbation effects from single-cell transcriptomic network structure, these results were interpreted as exploratory computational prioritization rather than direct evidence of causal gene regulation, protein-level signaling, or functional consequences.

### 2.11 Differential-expression and statistical analysis

Statistical analyses were structured to distinguish three analytical levels: donor-level comparisons, cell-level exploratory analyses, and pseudobulk differential-expression analyses.

First, donor-level analyses were used for all primary between-group comparisons of immune-cell composition and module-score distributions. Major immune-cell proportions, CD4^+^ T-cell state proportions, and module scores were calculated separately for each donor. Between-group comparisons were then performed using two-sided Wilcoxon rank-sum tests, with the donor treated as the biological unit. Exact P values were reported where applicable. For major immune-cell proportion analyses, major immune-cell populations not detected in individual donors were assigned a value of zero before donor-level comparison. These zero assignments were used only for donor-level composition analysis and were not interpreted as definitive biological absence of the corresponding cell population.

Second, cell-level differential-expression analyses were used primarily for cell-type annotation, transcriptional-state annotation, and exploratory visualization. Marker genes for major immune-cell populations, myeloid subclusters, granulocyte subclusters, unsupervised CD4^+^ T-cell clusters, and annotated CD4^+^ T-cell states were identified using Seurat-based Wilcoxon rank-sum tests. Genes were considered representative markers if they were expressed in at least 10% of cells in the corresponding cluster or state, had an average log_2_ fold change greater than 0.25, and had a Benjamini–Hochberg-adjusted P value below 0.05. Cell-level disease-group comparisons within the myeloid-cell and granulocyte compartments were performed only for exploratory visualization and hypothesis generation. These analyses were not interpreted as definitive disease-associated differential-expression results because individual cells from the same donor are not independent biological replicates.

Third, pseudobulk differential-expression analyses were performed as donor-level sensitivity analyses where sufficient donor representation was available. Raw counts were aggregated by donor within each annotated major immune-cell population or CD4^+^ T-cell transcriptional state, generating one pseudobulk profile per donor for each cell type or state. Pseudobulk comparisons were performed only when at least three donors per disease group contributed at least 10 cells to the corresponding population. Genes with low counts across pseudobulk samples were filtered before model fitting. Differential expression was tested using DESeq2, with disease group included as the main comparison variable and donor-level pseudobulk profiles treated as biological replicates. P values were adjusted using the Benjamini–Hochberg method.

## 3 Results

### 3.1 Quality control, integration, and major CSF immune-cell atlas

**Figure 1:**
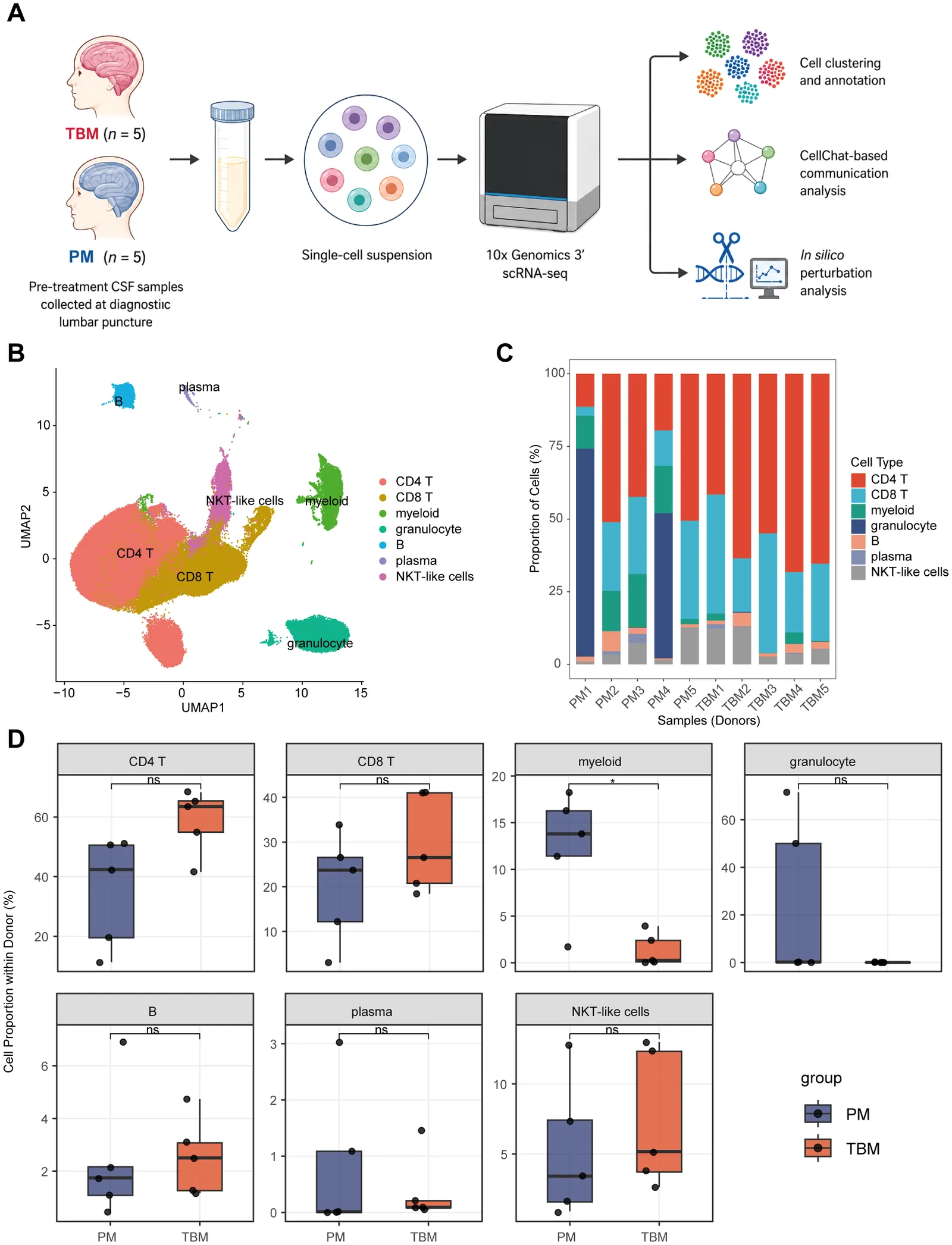
Study design and single-cell atlas of major cerebrospinal fluid immune-cell populations in tuberculous meningitis and purulent meningitis. **(A)** Schematic overview of the study design and analytical workflow. Pre-treatment CSF samples were collected during diagnostic lumbar puncture from patients with tuberculous meningitis (TBM, n = 5) and purulent meningitis (PM, n = 5). Fresh CSF immune cells were processed into single-cell suspensions and subjected to 10x Genomics 3′ single-cell RNA sequencing, followed by cell clustering and annotation, CellChat-based communication analysis, and exploratory in silico perturbation analysis. **(B)** UMAP visualization of annotated major CSF immune-cell populations, including CD4^+^ T cells, CD8^+^ T cells, myeloid cells, granulocytes, B cells, plasma cells, and NKT-like cells. **(C)** Donor-level stacked bar plot showing the proportional composition of major immune-cell populations in each CSF sample. **(D)** Donor-level comparison of major immune-cell proportions between PM and TBM samples. Each dot represents one donor. Box plots indicate the median and interquartile range, with whiskers extending to 1.5× the interquartile range. Between-group comparisons were performed using two-sided Wilcoxon rank-sum tests at the donor level. P values should be interpreted descriptively because of the limited donor number and multiple cell-type comparisons. ns, not significant; *P < 0.05.

We performed single-cell transcriptomic profiling of cerebrospinal fluid immune cells from five patients with tuberculous meningitis and five patients with purulent meningitis. The overall study design and analytical workflow are shown in **Figure 1A**. After sample-specific quality control, ambient RNA assessment, and doublet filtering, 96,594 cells were retained for downstream analyses.

The distributions of the number of detected genes per cell, unique molecular identifier counts per cell, mitochondrial transcript percentage, and scDblFinder doublet scores were broadly comparable across donors. Post-filtering scDblFinder classification showed that all cells retained in the final analysis object were labeled as singlets. However, because this classification was assessed after removal of high-confidence predicted doublets, it does not definitively exclude all residual multiplets.

In the pre-Harmony UMAP embedding, nFeature_RNA, nCount_RNA, and percent.mt did not show obvious spatial gradients that fully accounted for the major global transcriptional structure. Harmony correction subsequently improved mixing of cells from different donors while preserving broad immune-cell organization, as supported by donor- and disease-group-colored UMAP visualizations before and after integration and by cell-type and integration local inverse Simpson’s index assessments (cLISI and iLISI, respectively; **Supplementary Figure 1**).

The integrated UMAP resolved seven major immune-cell populations: CD4^+^ T cells, CD8^+^ T cells, myeloid cells, granulocytes, B cells, plasma cells, and natural killer T–like cells (**Figure 1**). Donor-level stacked bar plots demonstrated marked inter-individual variability in immune-cell composition in both disease groups. Several purulent meningitis donors exhibited granulocyte-rich or myeloid-enriched profiles, whereas several tuberculous meningitis donors showed relatively greater CD4^+^ T-cell representation.

Donor-level comparisons showed a nominally higher proportion of myeloid cells in purulent meningitis than in tuberculous meningitis. CD4^+^ T-cell proportions showed a non-significant tendency toward higher representation in tuberculous meningitis, whereas granulocyte proportions were numerically higher in purulent meningitis but did not reach statistical significance. No statistically significant between-group differences were observed for CD8^+^ T cells, B cells, plasma cells, or natural killer T–like cells.

### 3.2 Myeloid and granulocyte subclustering reveals heterogeneous innate immune-cell states

Compartment-specific reclustering resolved the initially annotated myeloid compartment into ten unsupervised subclusters, designated Myeloid_0 to Myeloid_9, and the granulocyte compartment into six subclusters, designated Gran_0 to Gran_5. The relative abundance of these subclusters varied substantially across donors, indicating marked inter-individual heterogeneity within the innate immune-cell compartment (**Supplementary Figure 2**).

Re-evaluation using coherent lineage-associated marker programs supported four broad transcriptomic identities: macrophage-like, monocyte-like, neutrophil-like, and CD1C^+^ dendritic cell–like populations. These annotations were supported by the spatial expression of C1QA, CD14, CSF3R, and CD1C, respectively, together with top-ranked marker genes across the revised identities (**Supplementary Figure 2**). These broad labels were used to describe transcriptomic identities and were not intended to imply functionally validated or mutually exclusive immune-cell subsets.

Marker-gene profiling identified coordinated T-cell-associated transcriptional programs in two originally defined myeloid subclusters. Myeloid_2 expressed T-cell-associated genes including IL7R, GZMK, LTB, TRAC, and CD3D, whereas Myeloid_5 expressed GZMA, CD3D, CD3E, TRAC, CCL5, GZMK, CD2, and IL32 (**Supplementary Figure 2**; **Supplementary Data S1**). These patterns involved multiple T-cell-lineage genes rather than isolated low-level TRAC expression. All cells retained in the final myeloid and granulocyte analysis object were labeled as singlets after doublet filtering. However, post-filtering singlet classification cannot definitively exclude residual heterotypic multiplets. Lymphocyte misclassification, lymphocyte contamination, and residual mixed droplets were therefore considered plausible explanations, while a contribution from ambient RNA carryover could not be excluded. Accordingly, Myeloid_2 and Myeloid_5 were not interpreted as bona fide myeloid states or as evidence of a novel TRAC-positive myeloid population.

Exploratory cell-level differential-expression analysis suggested relatively higher expression of antigen-presentation- and dendritic cell–associated genes, including HLA-DOB and CD1C, in TBM-derived myeloid cells. Granulocyte subclusters showed inflammatory and interferon-responsive transcriptional programs. Because individual cells derived from the same donor are not independent biological replicates, these cell-level comparisons were used for exploratory visualization and hypothesis generation and were not interpreted as definitive disease-associated differential-expression results.

To reduce the influence of cell-level pseudoreplication, donor-level pseudobulk differential-expression analysis was performed as a sensitivity analysis in populations with sufficient donor representation. Several candidate group-associated signals were identified within the myeloid compartment. TBM-associated myeloid signals included antigen-presentation- and dendritic cell–associated genes such as HLA-DOB, HLA-DQB2, HLA-DQA2, CLEC9A, DNASE1L3, and IDO2, whereas PM-associated signals included inflammatory granulocyte- and myeloid-associated genes such as S100A8, S100A12, and AQP9. In the granulocyte compartment, only a small number of genes reached a false discovery rate (FDR) threshold of <0.05, indicating limited donor-level transcriptional separation between TBM and PM. Complete pseudobulk differential-expression results are provided in **Supplementary Data S1**. Overall, these findings describe heterogeneous and donor-variable innate immune-cell transcriptional patterns rather than definitive disease-specific cellular states or signatures.

### 3.3 CD4^+^ T-cell subclustering identifies broad transcriptional states with donor-level variability

**Figure 2:**
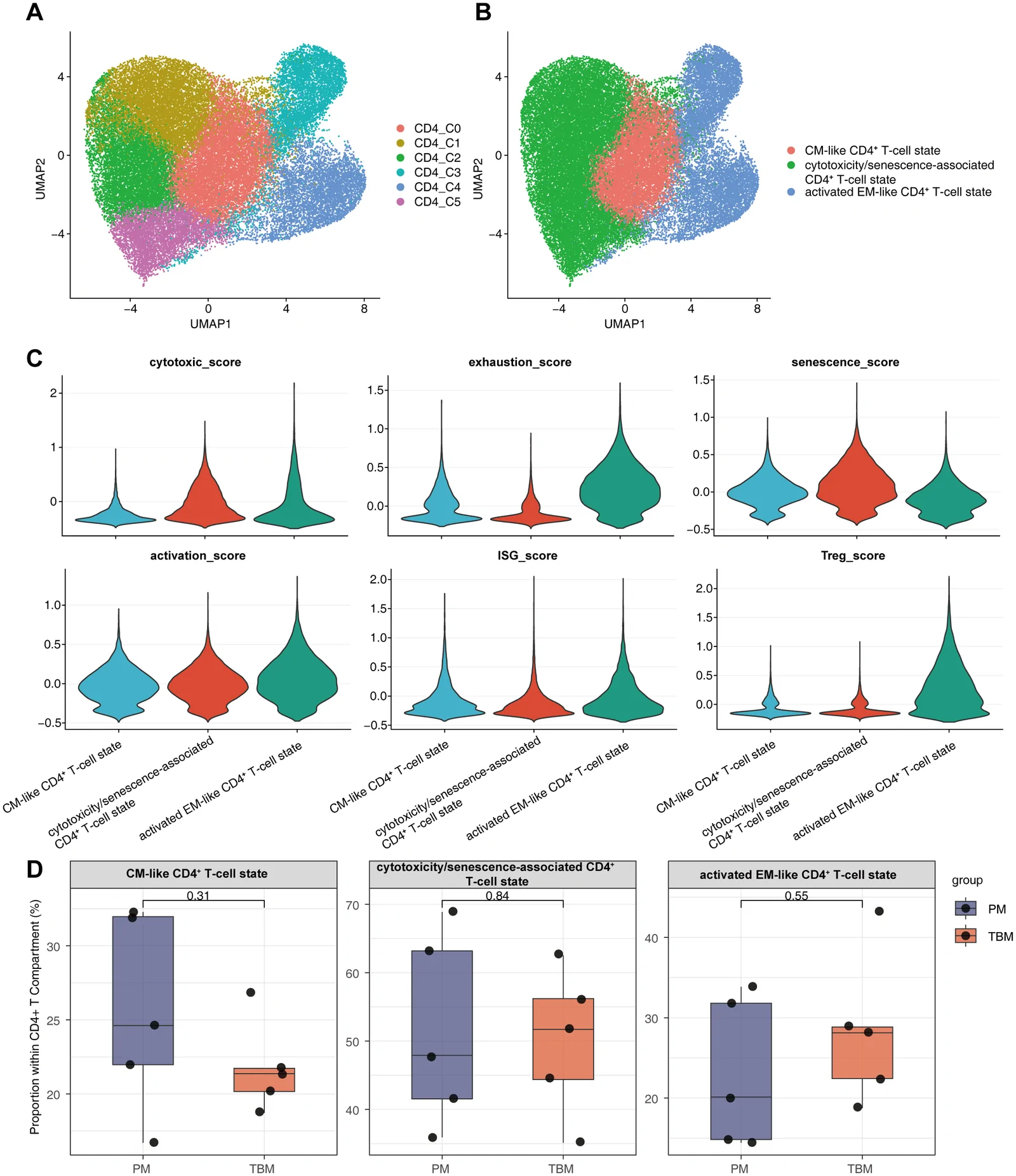
CD4^+^ T-cell subclustering identifies broad transcriptional states with donor-level variability in inflammatory cerebrospinal fluid. **(A)** UMAP visualization of unsupervised CD4^+^ T-cell clusters after reclustering of CD4^+^ T cells extracted from the integrated CSF single-cell dataset. Six unsupervised clusters were identified and denoted CD4_C0 to CD4_C5. **(B)** UMAP visualization of CD4^+^ T cells annotated into three broad transcriptional states based on UMAP structure, marker-gene expression, and module-score profiles: CM-like CD4^+^ T-cell state, cytotoxicity/senescence-associated CD4^+^ T-cell state, and activated EM-like CD4^+^ T-cell state. **(C)** Violin plots showing module-score distributions for cytotoxicity-, exhaustion-, senescence-, activation-, interferon-stimulated gene (ISG)-, and Treg-associated transcriptional programs across the three annotated CD4^+^ T-cell states. The cytotoxicity/senescence-associated state showed relatively higher senescence-associated features and selected cytotoxicity-associated signals, whereas the activated EM-like state showed higher activation-, ISG-, exhaustion-associated, and Treg-associated scores in subsets of cells. **(D)** Donor-level comparison of the proportions of the three CD4^+^ T-cell states between PM and TBM samples. Each dot represents one donor. Box plots indicate the median and interquartile range, with whiskers extending to 1.5× the interquartile range. Between-group comparisons were performed using two-sided Wilcoxon rank-sum tests at the donor level. No statistically significant differences were observed in the proportions of CM-like, cytotoxicity/senescence-associated, or activated EM-like CD4^+^ T-cell states between PM and TBM samples.

To characterize CD4^+^ T-cell heterogeneity within the inflammatory CSF microenvironment, CD4^+^ T cells were extracted from the integrated dataset and subjected to unsupervised reclustering. Six transcriptional clusters were initially identified and designated CD4_C0 to CD4_C5 (**Figure 2**). These clusters were distributed along a largely continuous UMAP landscape and did not form sharply separated cellular populations.

Comparison of cluster-specific marker-gene profiles showed that several of the six unsupervised clusters shared related characteristic genes and transcriptional programs rather than representing clearly distinct cellular identities. Clusters with concordant marker-gene expression and broadly similar module-score patterns were therefore consolidated into three broader transcriptional states to facilitate biological interpretation: a CM-like CD4^+^ T-cell state, a cytotoxicity/senescence-associated CD4^+^ T-cell state, and an activated EM-like CD4^+^ T-cell state (**Figure 2**). This consolidation represented a biologically guided descriptive summary, while the original six-cluster solution was retained as the primary computational output. Accordingly, the three broader states should be interpreted as related regions of a continuous transcriptional landscape rather than as mutually exclusive or functionally validated CD4^+^ T-cell subsets.

Module-score analysis revealed distinct but partially overlapping transcriptional programs across the three states. The cytotoxicity/senescence-associated state showed relatively higher senescence-associated scores together with selected cytotoxicity-associated features. In contrast, the activated EM-like state showed relatively greater activation-, exhaustion-, interferon-stimulated gene (ISG)-, and Treg-associated scores in subsets of cells. However, none of these programs was restricted to a single state, and substantial overlap was observed across the CD4^+^ T-cell landscape. Marker-level assessment similarly showed that selected cytotoxicity-, exhaustion-, and senescence-associated genes were expressed across multiple states rather than being uniformly confined to one discrete population.

Donor-level comparisons showed no statistically significant differences between PM and TBM samples in the proportions of the CM-like, cytotoxicity/senescence-associated, or activated EM-like CD4^+^ T-cell states (P = 0.31, P = 0.84, and P = 0.55, respectively). Additional UMAP visualizations colored by donor identity and disease group demonstrated broad intermixing of cells across the CD4^+^ T-cell transcriptional landscape, without a consistent donor-specific or disease-group-specific separation (**Supplementary Figure 3**). Donor-level comparisons of activation-, cytotoxicity-, exhaustion-, senescence-, ISG-, and Treg-associated module scores likewise showed no consistent differences between TBM and PM within the annotated CD4^+^ T-cell states.

### 3.4 MIF and CD74 expression across CSF immune-cell populations and CD4^+^ T-cell states

**Figure 3:**
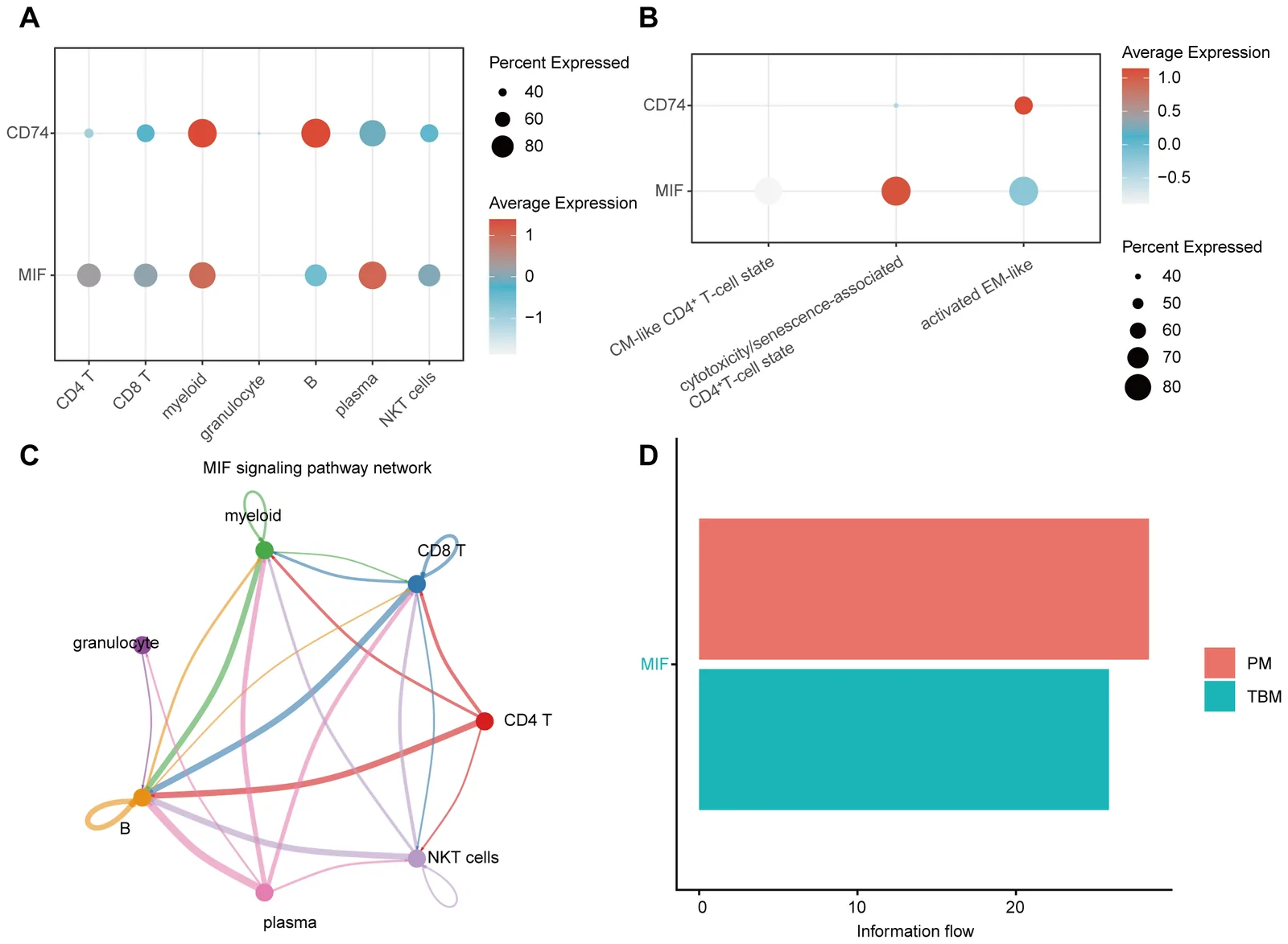
Multicompartment MIF and CD74 expression and CellChat-inferred MIF-related communication in inflammatory cerebrospinal fluid. **(A)** Dot plot showing MIF and CD74 expression across major cerebrospinal fluid (CSF) immune-cell populations, including CD4^+^ T cells, CD8^+^ T cells, myeloid cells, granulocytes, B cells, plasma cells, and NKT-like cells. Dot size indicates the percentage of cells expressing the indicated gene, and color represents scaled average expression. **(B)** Dot plot showing MIF and CD74 expression across the three annotated CD4^+^ T-cell transcriptional states, including CM-like, cytotoxicity/senescence-associated, and activated EM-like CD4^+^ T-cell states. Dot size indicates the percentage of expressing cells, and color represents scaled average expression. **(C)** CellChat-inferred MIF signaling pathway network among major CSF immune-cell populations. Nodes represent annotated immune-cell populations, and edges indicate inferred MIF-related communication between sender and receiver cell populations. Edge thickness reflects inferred communication strength. **(D)** Comparative information-flow analysis of the inferred MIF signaling pathway in PM and TBM samples. MIF-related signaling was detectable in both disease groups, but the overall inferred MIF information flow was not higher in TBM than in PM. These findings support the interpretation of MIF/CD74-related communication as a multicompartment inflammatory signaling program rather than a TBM-specific or CD4^+^ T-cell-restricted pathway.

We next examined MIF and CD74 expression across major CSF immune-cell populations. MIF was broadly detectable across multiple immune-cell compartments, including CD4^+^ T cells, CD8^+^ T cells, myeloid cells, B cells, plasma cells, and NKT-like cells. In contrast, CD74 expression was most prominent in antigen-presentation-associated compartments, particularly myeloid cells, B cells, and plasma cells, although detectable CD74 transcripts were also observed in selected T-cell populations. These expression patterns showed that MIF and CD74 signals in inflammatory CSF were distributed across multiple immune-cell compartments rather than being restricted to CD4^+^ T cells (**Figure 3**).

Within the CD4^+^ T-cell compartment, MIF was detectable across all three annotated transcriptional states, whereas CD74 expression was lower and more variable. Because CD74 is closely associated with antigen-presenting cell biology, detectable CD74 transcripts in CD4^+^ T cells were evaluated cautiously. We therefore assessed whether CD74-detectable CD4^+^ T cells retained T-cell lineage identity or showed evidence of potential technical artifacts, including ambient RNA contamination or doublet-related signals (**Supplementary Figure 4**).

Because CD74 expression was most evident in a subset of cytotoxicity/senescence-associated CD4^+^ T cells, CD74-detectable and CD74-undetectable cells within this state were compared. CD74-detectable cells retained canonical T-cell marker expression and did not show broad enrichment of B-cell, plasma-cell, myeloid-cell, or granulocyte markers. However, these cells showed higher predicted doublet scores than CD74-undetectable cells. Thus, CD74-detectable cells within this CD4^+^ T-cell state largely retained a T-cell transcriptional identity, but the increased doublet-related signal indicated that CD74 expression in these cells should be interpreted cautiously. Accordingly, detectable CD74 expression in CD4^+^ T cells were not considered definitive evidence of a functionally distinct CD74-expressing CD4^+^ T-cell subset.

### 3.5 CellChat analysis of MIF-related communication in inflammatory CSF

CellChat analysis was performed across major CSF immune-cell populations to explore transcriptome-inferred MIF-related communication in inflammatory CSF. The inferred global MIF-related signaling network involved multiple immune-cell compartments, including lymphoid, myeloid, B-cell, plasma-cell, and NKT-like populations. Comparative information-flow analysis showed that MIF-related signaling was detectable in both PM and TBM samples. However, the overall inferred MIF information flow was not higher in TBM than in PM, and pathway-level comparisons did not show selective enrichment of MIF-related signaling in TBM (**Figure 3**).

Comparative analysis of pathway-level communication networks further identified multiple immune-related signaling programs in CSF, including MHC-related, chemokine/cytokine-associated, adhesion-related, extracellular-matrix-associated, and immune-regulatory pathways. The complete pathway-level communication landscape is shown in **Supplementary Figure 5**. MIF-related communication was neither the highest-ranked pathway nor selectively enriched in TBM. It was selected for focused analysis as a biologically coherent representative axis linking broadly MIF-expressing immune populations with CD74-expressing antigen-presentation-associated populations. Accordingly, MIF–CD74 interactions were evaluated within the broader MIF-related CellChat output rather than as an isolated TBM-specific pathway. Together with the multicompartment expression patterns of MIF and CD74, these results indicate that MIF/CD74-related signals represent a transcriptome-inferred multicellular inflammatory communication program in CSF, rather than a CD4^+^ T-cell-restricted or TBM-specific interaction.

### 3.6 Exploratory *in silico* perturbation of MIF and CD74 in CSF immune cells

**Figure 4:**
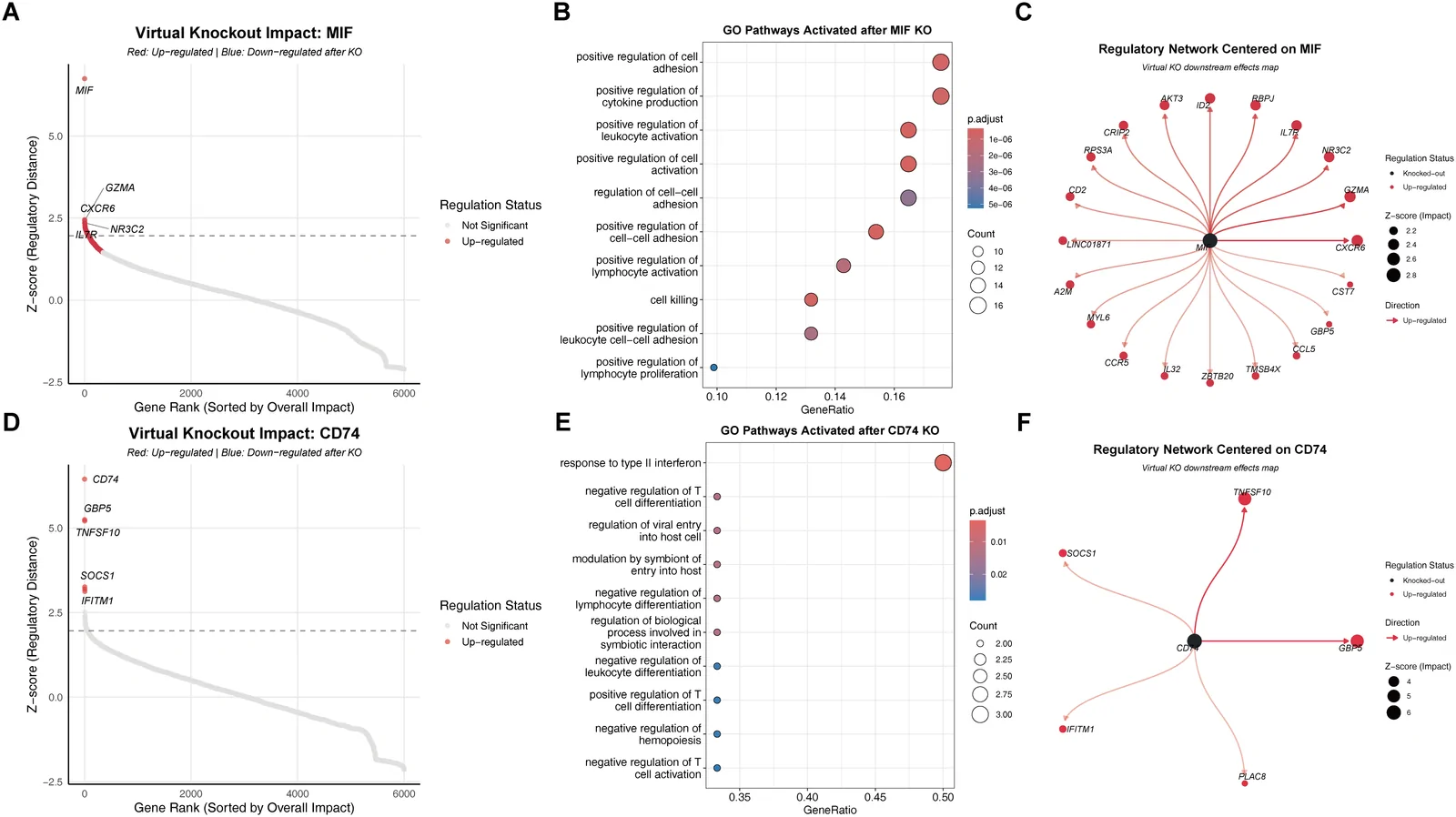
Exploratory *in silico* perturbation analysis of MIF and CD74 in cerebrospinal fluid immune cells. **(A)** Ranked regulatory-distance plot showing predicted transcriptional changes after virtual knockout of MIF in CD4^+^ T cells. Genes are ordered by overall predicted perturbation impact. The perturbation target MIF is shown as the reference gene, and selected genes with relatively large predicted regulatory-distance changes, including GZMA, CXCR6, IL7R, and NR3C2, are labeled. Red indicates genes predicted to be up-regulated after virtual knockout, whereas blue indicates genes predicted to be down-regulated after virtual knockout. **(B)** GO biological-process enrichment analysis of the predicted MIF perturbation-associated gene set in CD4^+^ T cells. Enriched terms included immune activation-related processes, such as regulation of cell adhesion, cytokine production, leukocyte activation, lymphocyte activation, and cell killing. Dot size indicates the number of genes included in each GO term, and color represents the adjusted P value. **(C)** Predicted perturbation-associated regulatory network centered on MIF after virtual knockout in CD4^+^ T cells. Nodes represent genes with predicted regulatory-distance changes, and edges indicate inferred network relationships. Node size reflects the predicted perturbation impact score. **(D)** Ranked regulatory-distance plot showing predicted transcriptional changes after virtual knockout of CD74 in myeloid cells. The perturbation target CD74 is shown as the reference gene, and selected genes with relatively large predicted regulatory-distance changes, including GBP5, TNFSF10, SOCS1, and IFITM1, are labeled. **(E)** GO biological-process enrichment analysis of the predicted CD74 perturbation-associated gene set in myeloid cells. Enriched terms included type II interferon response and immune-regulatory processes related to T-cell and lymphocyte differentiation. Dot size indicates the number of genes included in each GO term, and color represents the adjusted P value. **(F)** Predicted perturbation-associated regulatory network centered on CD74 after virtual knockout in myeloid cells. Nodes represent genes with predicted regulatory-distance changes, and edges indicate inferred network relationships. Node size reflects the predicted perturbation impact score. These in silico perturbation analyses were exploratory and should be interpreted as computational prioritization of MIF- and CD74-associated transcriptional programs rather than direct evidence of causal regulation, protein-level signaling, or functional consequences.

Exploratory in silico perturbation analyses were performed in selected CSF immune-cell compartments to prioritize transcriptional programs potentially associated with MIF and CD74. MIF was virtually perturbed in CD4^+^ T cells. In the regulatory-distance ranking, MIF was shown as the perturbation target. Genes with relatively large predicted regulatory-distance changes included GZMA, CXCR6, IL7R, and NR3C2 (**Figure 4**).

Gene Ontology enrichment analysis of the predicted MIF perturbation-associated gene set showed enrichment of immune activation-related biological processes. These included regulation of cell adhesion, cytokine production, leukocyte activation, lymphocyte activation, and cell killing. The predicted perturbation-associated network contained genes linked to T-cell activation, cytotoxicity-associated programs, chemokine receptor signaling, and inflammatory responses, including GZMA, CXCR6, IL7R, CCL5, IL32, CCR5, and A2M (**Figure 4**).

CD74 was virtually perturbed in myeloid cells. In the regulatory-distance ranking, CD74 was shown as the perturbation target. Genes with relatively large predicted regulatory-distance changes included GBP5, TNFSF10, SOCS1, and IFITM1. Gene Ontology enrichment analysis of the predicted CD74 perturbation-associated gene set showed enrichment of immune-regulatory and interferon-associated biological processes, including type II interferon response and regulation of T-cell or lymphocyte differentiation. The predicted perturbation-associated network contained interferon-responsive and immune-regulatory genes, including GBP5, TNFSF10, SOCS1, IFITM1, and PLAC8 (**Figure 4**).

## 4 Discussion

In this study, we generated a single-cell transcriptomic atlas of pre-treatment CSF immune cells from patients with TBM and PM. The analysis showed marked inter-individual heterogeneity in the inflammatory CSF immune landscape. PM samples showed nominally higher myeloid-cell representation and numerically higher granulocyte representation, whereas TBM samples showed a non-significant trend toward higher CD4^+^ T-cell contribution. Within the CD4^+^ T-cell compartment, cells formed a continuous transcriptional landscape rather than sharply separated functional subsets. The annotated CD4^+^ T-cell transcriptional states did not show consistent donor-level disease-group-associated expansion. MIF and CD74 were detected across multiple immune-cell compartments, and CellChat analysis supported MIF/CD74-related signaling as a transcriptome-inferred multicellular inflammatory communication program rather than a TBM-specific or CD4^+^ T-cell-restricted pathway.

The global immune-cell differences between TBM and PM are consistent with the distinct biological features of these two forms of meningitis. PM usually represents an acute bacterial inflammatory process. Innate immune-cell recruitment, especially involving granulocytes and myeloid cells, is a major component of the CSF response in bacterial meningitis (Koedel et al., 2002; van de Beek et al., 2006; Mook-Kanamori et al., 2011). In contrast, TBM usually follows a more subacute or chronic course. It is characterized by persistent mycobacterial antigenic stimulation, blood–brain barrier disruption, meningeal inflammation, vasculopathy, and coordinated innate and adaptive immune responses (Rock et al., 2008; O’Garra et al., 2013; Wilkinson et al., 2017; Rohlwink et al., 2019). In the present dataset, donor-level analysis showed higher myeloid-cell proportions in PM and a non-significant trend toward higher CD4^+^ T-cell representation in TBM. These findings support broad compositional differences between the two inflammatory meningitis conditions. They also highlight the importance of donor-level analysis rather than pooled-cell interpretation in small clinical single-cell cohorts.

To place the CD4^+^ T-cell findings within the broader CSF immune landscape, we also examined the myeloid and granulocyte compartments. Myeloid cells showed heterogeneous transcriptional states, including monocyte/macrophage-like, antigen-presentation-associated, dendritic cell-like, and inflammatory features. Granulocytes also showed variable donor-level composition and transcriptional programs related to inflammatory and interferon-responsive features. These observations are consistent with previous evidence that TBM immunopathogenesis involves compartmentalized and multicellular immune responses within the CNS and CSF, rather than a single dominant leukocyte population (Rock et al., 2008; O’Garra et al., 2013; Rohlwink et al., 2019). In the present study, the myeloid and granulocyte analyses provide a broader cellular context for interpreting adaptive immune-cell findings. Because the compartment-specific differential-expression analyses were exploratory and influenced by donor-level heterogeneity, they should be interpreted as descriptive evidence of innate immune-cell diversity rather than definitive disease-specific transcriptional signatures.

At the CD4^+^ T-cell level, our findings support a conservative transcriptional-state interpretation. After reclustering, marker-level assessment, module-score analysis, and donor-level comparison, CSF CD4^+^ T cells were distributed along a continuous transcriptional landscape. They did not form sharply separated functional subsets. The annotated CM-like, cytotoxicity/senescence-associated, and activated EM-like CD4^+^ T-cell states showed partially overlapping transcriptional programs. None showed consistent disease-group-associated expansion at the donor level. The cytotoxicity/senescence-associated state displayed selected cytotoxicity- and senescence-associated features. However, canonical cytotoxic effector molecules and exhaustion-associated markers were not uniformly enriched within a single discrete CD4^+^ T-cell state.

This distinction is important. Granzyme-associated transcripts or inhibitory receptor-associated transcripts alone do not establish cytotoxic function, proliferative impairment, cytokine dysfunction, or bona fide T-cell exhaustion. Prior studies in tuberculosis have reported cytotoxicity-related, granulysin-associated, PD-1-associated, and exhaustion-like transcriptional or functional features in T-cell populations during active disease (Barber et al., 2011; Day et al., 2011; Jayaraman et al., 2016; Balin et al., 2018). Our data are compatible with the presence of such programs in inflammatory CSF. However, the current dataset does not demonstrate functional cytotoxicity or true T-cell exhaustion. Accordingly, CSF CD4^+^ T cells are better interpreted as overlapping transcriptional states involving activation-, cytotoxicity-, senescence-, exhaustion-, ISG-, and Treg-associated programs, with substantial donor-level variability.

The MIF/CD74 analysis illustrates how a single-cell atlas can be used to examine candidate signaling pathways in a multicellular context. The results did not support a TBM-specific or CD4^+^ T-cell-restricted MIF–CD74 pathway. Instead, MIF and CD74 were distributed across multiple immune-cell compartments. MIF was detectable across lymphoid and myeloid populations, whereas CD74 expression was more prominent in antigen-presentation-associated compartments, including myeloid cells, B cells, and plasma cells. This distribution is biologically plausible. MIF is a pleiotropic inflammatory cytokine, and CD74 functions both as an MHC class II-associated molecule and as a receptor component for MIF-related signaling (Calandra and Roger, 2003; Leng et al., 2003; Shi et al., 2006; Das et al., 2013; Farr et al., 2020). Within CD4^+^ T cells, CD74 transcripts were detectable in selected cells. However, CD74-detectable cells showed higher predicted doublet scores. Although these cells retained detectable T-cell marker expression, the increased doublet-related signal requires caution. Therefore, detectable CD74 expression within CD4^+^ T-cell states were interpreted cautiously at the transcriptomic level and was not considered evidence of a distinct functional CD74-expressing CD4^+^ T-cell subset.

CellChat analysis detected MIF-related communication across multiple CSF immune-cell populations and in both disease groups. The overall inferred MIF information flow was not selectively increased in TBM compared with PM. Pathway-level comparisons also showed that MIF-related signaling was one component of a broader inferred communication network. This network included MHC-related, chemokine/cytokine-associated, adhesion-related, extracellular-matrix-associated, and immune-regulatory pathways. Because CellChat infers communication from transcript abundance and curated ligand–receptor relationships, these findings should be interpreted as transcriptome-inferred candidate interactions rather than direct evidence of protein-level ligand–receptor binding or downstream pathway activation (Jin et al., 2021). The MIF–CD74 ligand–receptor pair remains biologically relevant because of the known roles of MIF and CD74 in inflammation, antigen presentation, and immune-cell regulation (Leng et al., 2003; Shi et al., 2006; Farr et al., 2020). In this dataset, however, MIF/CD74-related signals are best viewed as part of a shared multicellular inflammatory CSF communication program.

The exploratory in silico perturbation analyses provided computational prioritization of MIF- and CD74-associated transcriptional programs. Virtual perturbation of MIF in CD4^+^ T cells was associated with predicted regulatory-distance changes involving genes related to T-cell activation, cytotoxicity-associated molecules, chemokine receptors, and inflammatory mediators. Virtual perturbation of CD74 in myeloid cells was associated with predicted regulatory-distance changes involving interferon-responsive and immune-regulatory genes. These results are consistent with the broad immune-regulatory context in which MIF and CD74 were detected. Nevertheless, scTenifoldKnk is a network-based computational method. It estimates transcriptional effects from single-cell gene regulatory networks and cannot establish causality, directionality of regulation, protein-level signaling, or functional consequences (Osorio et al., 2022).

The interferon-related signals observed in the perturbation and innate immune-cell analyses also require cautious interpretation. Interferon-associated transcriptional programs are frequently observed in active tuberculosis, including neutrophil-driven and type I interferon-related signatures (Berry et al., 2010; Moreira-Teixeira et al., 2018). Their biological effects are context dependent. They may reflect pathogen-driven inflammation, host defense, or immunopathology depending on disease stage, tissue compartment, and cell type (Berry et al., 2010; O’Garra et al., 2013; Moreira-Teixeira et al., 2018). In the present CSF dataset, interferon-associated genes appeared in exploratory perturbation and innate immune-cell analyses. These observations do not establish whether interferon-related programs are protective, pathogenic, or compensatory in TBM.

Clinically, the present findings provide a descriptive and hypothesis-generating framework. They do not provide direct evidence for therapeutic targeting. Current TBM treatment remains centered on anti-tuberculosis chemotherapy and adjunctive anti-inflammatory therapy. Adjunctive dexamethasone has been shown to improve survival in selected HIV-negative patients with TBM, although neurological outcomes remain variable (Thwaites et al., 2004). More recent data suggest that adjunctive dexamethasone does not provide the same survival benefit in HIV-positive adults with TBM (Donovan et al., 2023). Other host-directed strategies, such as adjunctive aspirin, have shown signals that warrant further evaluation but remain insufficient to establish routine practice (Mai et al., 2018). Host-directed therapy in TBM requires careful balancing of antimicrobial immunity and inflammation-mediated CNS injury (Davis and Wilkinson, 2021). The present transcriptomic findings provide a candidate basis for future investigation of MIF/CD74-related communication in inflammatory CSF. They are not sufficient to define MIF or CD74 as therapeutic targets. Such a conclusion would require protein-level validation, functional signaling assays, and assessment of effects on pathogen control, neuroinflammation, immune-cell function, and CNS safety.

Several clinical and methodological limitations should be considered. First, the cohort was small, and all between-group comparisons should be interpreted as exploratory. Second, the TBM and PM groups differed in age and selected CSF inflammatory parameters. Age-related immune remodeling may influence T-cell activation, senescence-associated transcriptional programs, and innate immune-cell composition. Third, PM is biologically heterogeneous. Donor-level variation in CSF leukocyte counts, cytokine concentrations, pathogen type, and inflammatory intensity may have contributed to the observed immune-cell composition. To mitigate pseudoreplication, we used donor-level summaries for cell-composition and module-score comparisons. This strategy is consistent with recommendations for multi-sample single-cell studies, in which the donor should be treated as the biological replicate (Crowell et al., 2020; Squair et al., 2021; Zimmerman et al., 2021). Nevertheless, the sample size was insufficient for robust adjustment of age, disease duration, pathogen type, and inflammatory severity. Fourth, single-cell RNA sequencing measures transcript abundance. It does not directly establish protein expression, ligand–receptor binding, downstream pathway activation, or cellular function. CellChat and in silico perturbation analyses therefore provide computationally inferred candidate interactions rather than mechanistic proof. Fifth, the available transcriptomic and quality-control outputs could not definitively distinguish among lymphocyte misclassification, ambient RNA carryover, and residual heterotypic multiplets in the two original myeloid subclusters expressing coordinated T-cell-associated genes. Stepwise per-sample doublet-removal counts were not available, and post-filtering classification of retained cells as singlets cannot exclude all residual multiplets. In addition, post-Harmony technical-covariate overlays were unavailable. These limitations constrain definitive attribution of the cross-lineage transcripts and should be addressed in future studies using prospectively archived quality-control outputs and orthogonal protein-level cell-identity assessment. Finally, the lack of independent validation by flow cytometry, CSF protein measurement, spatial analysis, or functional assays limits causal interpretation of the MIF/CD74-related findings.

In conclusion, this study provides a donor-aware single-cell transcriptomic atlas of the inflammatory CSF immune microenvironment in TBM and PM. The data highlight substantial inter-individual heterogeneity, a PM-associated tendency toward myeloid- and granulocyte-rich inflammatory composition, and overlapping CD4^+^ T-cell transcriptional states that do not define a consistently expanded TBM-specific functional subset. MIF and CD74 were distributed across multiple immune-cell compartments, and CellChat analysis supported MIF/CD74-related communication as a shared multicellular inflammatory program rather than a TBM-specific or CD4^+^ T-cell-restricted pathway. These findings provide a framework for future validation of candidate CSF immune programs in larger longitudinal cohorts using protein-level, spatial, and functional approaches.

## Data Availability

The single-cell RNA sequencing data presented in this study have been deposited in the NCBI BioProject repository under accession number PRJNA1501896.
