# Supplementary material for: Single-cell transcriptomic profiling of cerebrospinal fluid reveals immune-cell heterogeneity and multicompartment MIF/CD74-related communication in tuberculous and purulent meningitis
Source: Front Cell Infect Microbiol. 2026 Aug 13;16:1867160. doi: 10.3389/fcimb.2026.1867160 (PMC13518202; doi:10.3389/fcimb.2026.1867160)
Supplement: Supplementary file 1 [file Table1.docx]

**Supplementary Figure S1. Quality-control metrics, doublet assessment, and Harmony integration performance of the cerebrospinal fluid single-cell RNA-sequencing dataset.**
(A–D) Violin plots showing cell-level quality-control metrics across individual donors, including the number of detected genes per cell (nFeature_RNA), unique molecular identifier counts per cell (nCount_RNA), the percentage of mitochondrial transcripts (percent.mt), and scDblFinder-predicted doublet scores, respectively.
(E) UMAP visualization of the final analysis object after doublet filtering, coloured by post-filtering scDblFinder classification. All retained cells were labelled as singlets. Because the classification is shown after removal of high-confidence predicted doublets, this panel does not display the original spatial distribution of predicted doublets and does not exclude all residual multiplets.
(F–H) The pre-Harmony global UMAP embedding coloured by nFeature_RNA, nCount_RNA, and percent.mt, respectively.
(I) Comparison of the local inverse Simpson’s index (LISI) before and after Harmony integration. Cell-type LISI (cLISI) was used to assess preservation of biological cell-type structure, whereas integration LISI (iLISI) was used to evaluate mixing across donors. Higher iLISI values indicate improved donor mixing, while low and relatively stable cLISI values indicate preservation of cell-type separation.
(J, K) UMAP visualizations colored by donor identity before and after Harmony integration, respectively.
(L, M) UMAP visualizations colored by disease group before and after Harmony integration, respectively.

**Supplementary Figure S2. Composition, marker-gene profiles, canonical immune-cell reannotation, and technical quality assessment of myeloid and granulocyte populations in cerebrospinal fluid.**
(A) Donor-level composition of the initially identified myeloid subclusters (Myeloid_0–Myeloid_9). Each stacked bar represents one donor, and the proportions of all subclusters sum to 100% within each sample.
(B) Dot plot showing representative marker genes across the initially identified myeloid subclusters. Dot size indicates the percentage of cells expressing each gene, and color represents scaled average expression.
(C) Volcano plot showing differential gene expression in myeloid cells between tuberculous meningitis (TBM) and purulent meningitis (PM). The x-axis represents the average log₂ fold change, and the y-axis represents the −log₁₀ adjusted P value. Red and blue points indicate genes relatively upregulated in TBM and PM, respectively, whereas gray points indicate genes that did not meet the prespecified differential-expression criteria. Selected representative genes are labeled.
(D) UMAP visualization of the revised broad transcriptomic annotation of the innate immune-cell compartment, showing macrophage-like, monocyte-like, neutrophil-like, and CD1C⁺ dendritic-cell-like populations.
(E) Donor-level composition of the initially identified granulocyte subclusters (Gran_0–Gran_5).
(F) Dot plot showing representative marker genes across the initially identified granulocyte subclusters. Dot size indicates the percentage of cells expressing each gene, and color represents scaled average expression.
(G) Volcano plot showing differential gene expression in granulocytes between TBM and PM. The x-axis represents the average log₂ fold change, and the y-axis represents the −log₁₀ adjusted P value. Red and blue points indicate genes relatively upregulated in TBM and PM, respectively, whereas gray points indicate genes that did not meet the prespecified differential-expression criteria. Selected representative genes are labeled.
(H) Feature plots showing canonical lineage markers used to support the revised annotation, including CD14 for monocytes, C1QA for macrophages, CSF3R for neutrophils, and CD1C for dendritic cells. Color intensity represents gene-expression level.
(I) Dot plot showing top-ranked differentially expressed marker genes across the four revised innate immune-cell identities. Dot size indicates the percentage of cells expressing each gene, and color represents scaled average expression.
(J) Pooled proportional composition of the four revised innate immune-cell identities in the PM and TBM groups. This panel provides a descriptive summary based on all cells within each disease group and was not used for donor-level statistical inference.
(K) Violin plots showing the distributions of scDblFinder doublet scores, the number of detected genes per cell (nFeature_RNA), and unique molecular identifier counts per cell (nCount_RNA) across the revised cell identities.
(L) UMAP visualization of the myeloid and granulocyte compartment after doublet filtering, colored by doublet classification. All cells retained for downstream analyses were classified as singlets.

**Supplementary Figure S3. Validation and donor-level module-score assessment of CD4⁺ T-cell transcriptional states in inflammatory cerebrospinal fluid.**
(A) UMAP visualization of reclustered CD4⁺ T cells colored by individual donor, showing broad intermixing of cells from different donors across the CD4⁺ T-cell transcriptional landscape.
(B) UMAP visualization of reclustered CD4⁺ T cells colored by disease group, showing that the annotated CD4⁺ T-cell states were not driven by complete separation between purulent meningitis (PM) and tuberculous meningitis (TBM) samples.
(C) Violin plots showing the expression of selected cytotoxicity-associated genes across the three annotated CD4⁺ T-cell transcriptional states, including GZMA, GZMK, NKG7, CTSW, KLRK1, CCL5, GZMB, PRF1, GNLY, and CST7.
(D) Violin plots showing the expression of selected exhaustion- and senescence-associated genes across the three annotated CD4⁺ T-cell transcriptional states, including PDCD1, LAG3, HAVCR2, TIGIT, TOX, CTLA4, ENTPD1, KLRG1, DPP4, and A2M.
(E–J) Donor-level comparisons of module scores for activation-, cytotoxicity-, exhaustion-, interferon-stimulated gene (ISG)-, senescence-, and Treg-associated transcriptional programs across the three annotated CD4⁺ T-cell states. Each dot represents one donor. Box plots indicate the median and interquartile range, with whiskers extending to 1.5× the interquartile range. Between-group comparisons were performed using two-sided Wilcoxon rank-sum tests at the donor level. P values should be interpreted descriptively because of the limited donor number and multiple module-score comparisons.

**Supplementary Figure S4. Assessment of lineage identity and potential technical artifacts in CD74-detectable CD4⁺ T cells.**
(A) Dot plot comparing lineage and contamination-associated marker expression between CD74-undetectable (CD74⁻) and CD74-detectable (CD74⁺) cells within the cytotoxicity/senescence-associated CD4⁺ T-cell state. T-cell markers included CD3D, CD3E, TRAC, and CD4; cytotoxicity-associated markers included GZMA, GZMK, NKG7, CTSW, and KLRK1; B-cell and plasma-cell markers included MS4A1, CD79A, MZB1, JCHAIN, and IGKC; myeloid and granulocyte-associated markers included LYZ, CD14, S100A8, S100A9, and CSF3R. Dot size indicates the percentage of cells expressing the indicated gene, and color represents scaled average expression.
(B) Violin plots showing representative marker expression in CD74⁻ and CD74⁺ cells. CD74⁺ cells retained expression of canonical T-cell markers, including CD3E and CD4, and showed expression of selected cytotoxicity-associated genes, whereas broad enrichment of B-cell marker CD79A or myeloid marker LYZ was not observed.
(C) Comparison of predicted doublet scores between CD74⁻ and CD74⁺ cells. Violin plots with embedded box plots show the distribution of doublet scores. The P value was calculated using a two-sided Wilcoxon rank-sum test. CD74⁺ cells showed higher predicted doublet scores than CD74⁻ cells.

**Supplementary Figure S5. Comparative information-flow analysis of CellChat-inferred signaling pathways in inflammatory cerebrospinal fluid.**
Bar plot showing the relative information flow of CellChat-inferred signaling pathways between purulent meningitis (PM) and tuberculous meningitis (TBM) samples. Each row represents one inferred signaling pathway, and the horizontal bar indicates the relative contribution of PM and TBM to the total inferred information flow for that pathway. The vertical dashed line at 0.5 indicates equal relative contribution between the two disease groups. Pathways with larger PM-associated relative information flow are shown toward the upper part of the plot, whereas pathways with larger TBM-associated relative information flow are shown toward the lower part of the plot.
