# Supplementary material for: Single-cell transcriptomic profiling of cerebrospinal fluid reveals immune-cell heterogeneity and multicompartment MIF/CD74-related communication in tuberculous and purulent meningitis
Source: Front Cell Infect Microbiol. 2026 Aug 13;16:1867160. doi: 10.3389/fcimb.2026.1867160 (PMC13518202; doi:10.3389/fcimb.2026.1867160)
Supplement: Supplementary file 2 [file Table2.docx]

**Supplementary Table S1. Clinical and cerebrospinal fluid characteristics of patients with tuberculous meningitis and purulent meningitis**

| **Characteristics** | **TBM group (n = 5)** | **PM group (n = 5)** | **P value** |
| --- | --- | --- | --- |
| Age, years | 67.0 [61.0-69.0] | 56.0 [47.0-57.0] | 0.036 |
| Male sex, n (%) | 3 (60.0) | 3 (60.0) | >0.999 |
| Pathogen detected in CSF, n (%) | 5 (100.0) | 5 (100.0) | - |
| *Mycobacterium tuberculosis*, n (%) | 5 (100.0) | 0 (0.0) | - |
| *Streptococcus pneumoniae*, n (%) | 0 (0.0) | 4 (80.0) | - |
| *Streptococcus* spp., n (%) | 0 (0.0) | 1 (20.0) | - |
| Meningeal irritation signs, n (%) | 2 (40.0) | 4 (80.0) | 0.524 |
| Headache and/or fever, n (%) | 3 (60.0) | 1 (20.0) | 0.524 |
| Any clinical treatment before CSF sampling, n (%) | 0 (0.0) | 0 (0.0) | - |
| Glucocorticoid use before CSF sampling, n (%) | 0 (0.0) | 0 (0.0) | - |
| Underlying diseases/comorbidities, n (%) | 0 (0.0) | 0 (0.0) | - |
| CSF white blood cells, x10^6/L | 81.0 [72.0-85.0] | 202.0 [168.0-276.0] | 0.055 |
| CSF glucose, mmol/L | 2.8 [2.7-3.0] | 2.3 [2.3-2.4] | 0.015 |
| CSF protein, mg/L | 1212.0 [541.0-1249.0] | 1090.0 [364.0-1100.0] | 0.548 |
| IL-1, pg/mL | 30.0 [26.0-47.0] | 7.0 [5.0-37.0] | 0.400 |
| IL-2, pg/mL | 314.0 [194.0-812.0] | 146.0 [78.0-180.0] | 0.151 |
| IL-6, pg/mL | 30.0 [26.0-47.0] | 990.0 [260.0-1000.0] | 0.036 |
| IL-8, pg/mL | 169.0 [89.0-691.0] | 540.0 [107.0-690.0] | 1.000 |
| IL-10, pg/mL | 15.0 [14.0-28.0] | 20.0 [12.0-37.0] | 0.917 |
| TNF-alpha, pg/mL | 30.0 [30.0-32.0] | 15.0 [13.0-21.0] | 0.402 |

Note. Data are presented as median [interquartile range] or n (%). Between-group comparisons were performed using the Wilcoxon rank-sum test for continuous variables and Fisher’s exact test for categorical variables, where appropriate. Given the limited sample size, P values are provided for descriptive purposes and should not be interpreted as definitive evidence of group-level differences. TBM, tuberculous meningitis; PM, purulent meningitis; CSF, cerebrospinal fluid; IL, interleukin; TNF-α, tumor necrosis factor-α.

**Supplementary Table S2. Individual-level clinical characteristics of included patients**

| **Diagnosis/group** | **Sample ID** | **Sex** | **Age, years** | **CSF pathogen detected** | **Main clinical manifestations** | **Treatment before CSF sampling** | **Glucocorticoid use before CSF sampling** | **Underlying diseases/comorbidities** |
| --- | --- | --- | --- | --- | --- | --- | --- | --- |
| PM | CSF.data1 | Male | 57 | *Streptococcus pneumoniae* | Meningeal irritation signs | None | No | None |
| PM | CSF.data2 | Male | 44 | *Streptococcus pneumoniae* | Headache and fever | None | No | None |
| PM | CSF.data3 | Male | 66 | *Streptococcus pneumoniae* | Meningeal irritation signs | None | No | None |
| PM | CSF.data4 | Female | 56 | *Streptococcus* spp. | Meningeal irritation signs | None | No | None |
| PM | CSF.data5 | Female | 47 | *Streptococcus pneumoniae* | Meningeal irritation signs | None | No | None |
| TBM | CSF.data6 | Male | 67 | *Mycobacterium tuberculosis* | Headache | None | No | None |
| TBM | CSF.data7 | Male | 61 | *Mycobacterium tuberculosis* | Headache | None | No | None |
| TBM | CSF.data8 | Male | 69 | *Mycobacterium tuberculosis* | Meningeal irritation signs | None | No | None |
| TBM | CSF.data9 | Female | 58 | *Mycobacterium tuberculosis* | Headache | None | No | None |
| TBM | CSF.data10 | Female | 69 | *Mycobacterium tuberculosis* | Meningeal irritation signs | None | No | None |

Note. Sample labels are de-identified identifiers used for the single-cell RNA-seq analysis. Treatment before CSF sampling refers to antimicrobial, antituberculous, glucocorticoid, immunosuppressive, or other disease-modifying treatment administered before diagnostic CSF collection. TBM, tuberculous meningitis; PM, purulent meningitis; CSF, cerebrospinal fluid.

**Supplementary Table S3. Individual-level CSF laboratory and cytokine findings**

| **Sample ID** | **White blood cells, x10^6/L** | **Glucose, mmol/L** | **Protein, mg/L** | **IL-1, pg/mL** | **IL-2, pg/mL** | **IL-6, pg/mL** | **IL-8, pg/mL** | **IL-10, pg/mL** | **TNF-alpha, pg/mL** |
| --- | --- | --- | --- | --- | --- | --- | --- | --- | --- |
| CSF.data1 | 1567 | 2.2 | 1090 | 99 | 146 | 1000 | 690 | 112 | 59 |
| CSF.data2 | 202 | 2.5 | 364 | 5 | 78 | 260 | 107 | 12 | 13 |
| CSF.data3 | 276 | 2.3 | 2508 | 5 | 4355 | 1000 | 1645 | 20 | 15 |
| CSF.data4 | 168 | 2.3 | 1100 | 37 | 180 | 990 | 540 | 37 | 21 |
| CSF.data5 | 99 | 2.4 | 221 | 7 | 50 | 37 | 54 | 5 | 7 |
| CSF.data6 | 230 | 2.8 | 1249 | 47 | 182 | 97 | 709 | 54 | 30 |
| CSF.data7 | 72 | 2.5 | 425 | 30 | 314 | 30 | 691 | 28 | 32 |
| CSF.data8 | 81 | 3.4 | 541 | 8 | 194 | 8 | 64 | 5 | 12 |
| CSF.data9 | 60 | 2.7 | 1212 | 47 | 812 | 47 | 89 | 15 | 39 |
| CSF.data10 | 85 | 3.0 | 1326 | 26 | 1007 | 26 | 169 | 14 | 30 |

Note. All laboratory and cytokine values were measured in cerebrospinal fluid. Sample labels correspond to the donor identifiers used in the single-cell RNA-seq analysis. CSF, cerebrospinal fluid; IL, interleukin; PM, purulent meningitis; TBM, tuberculous meningitis; TNF-α, tumor necrosis factor-α.

**Supplementary Table S4. Per-sample sequencing and post-quality-control cell-count summary**

| **Sample label** | **Diagnosis** | **Raw cells called by Cell Ranger** | **Final analyzed singlets** | **Annotated major immune cells** | **Other / unassigned cells** |
| --- | --- | --- | --- | --- | --- |
| PM1 | PM | 8,935 | 8,184 | 8,181 | 3 |
| PM2 | PM | 8,810 | 8,020 | 8,012 | 8 |
| PM3 | PM | 22,612 | 15,776 | 13,488 | 2,288 |
| PM4 | PM | 9,896 | 8,341 | 8,334 | 7 |
| PM5 | PM | 9,663 | 8,894 | 8,893 | 1 |
| TBM1 | TBM | 9,235 | 8,244 | 8,182 | 62 |
| TBM2 | TBM | 11,972 | 10,806 | 10,806 | 0 |
| TBM3 | TBM | 10,812 | 9,839 | 9,839 | 0 |
| TBM4 | TBM | 12,047 | 10,782 | 10,737 | 45 |
| TBM5 | TBM | 8,376 | 7,708 | 7,707 | 1 |
| **Total** | **-** | **112,358** | **96,594** | **94,179** | **2,415** |

Note. Raw cell calls by Cell Ranger refer to the initial cell calls generated from the Cell Ranger pipeline. Final analyzed singlets refer to post-quality-control singlet cells retained after filtering, ambient RNA assessment, and doublet removal. Annotated major immune cells refer to cells assigned to the seven major immune-cell categories used for donor-level composition analysis. Other/unassigned cells were retained in the post-quality-control object but were not assigned to these major immune-cell categories. PM, purulent meningitis; TBM, tuberculous meningitis; QC, quality control.

**Supplementary Table S5. Donor-level counts and proportions of major CSF immune-cell populations**

*A. PM donors*

| **Sample label** | **Diagnosis** | **Cell type** | **Cell count** | **Proportion of annotated major immune cells (%)** |
| --- | --- | --- | --- | --- |
| PM1 | PM | CD4 T | 929 | 11.36 |
| PM1 | PM | CD8 T | 249 | 3.04 |
| PM1 | PM | myeloid | 935 | 11.43 |
| PM1 | PM | granulocyte | 5,850 | 71.51 |
| PM1 | PM | B | 143 | 1.75 |
| PM1 | PM | plasma | 0 | 0.00 |
| PM1 | PM | NKT cells | 75 | 0.92 |
| PM2 | PM | CD4 T | 4,091 | 51.06 |
| PM2 | PM | CD8 T | 1,900 | 23.71 |
| PM2 | PM | myeloid | 1,106 | 13.80 |
| PM2 | PM | granulocyte | 2 | 0.02 |
| PM2 | PM | B | 552 | 6.89 |
| PM2 | PM | plasma | 87 | 1.09 |
| PM2 | PM | NKT cells | 274 | 3.42 |
| PM3 | PM | CD4 T | 5,715 | 42.37 |
| PM3 | PM | CD8 T | 3,581 | 26.55 |
| PM3 | PM | myeloid | 2,462 | 18.25 |
| PM3 | PM | granulocyte | 29 | 0.22 |
| PM3 | PM | B | 292 | 2.16 |
| PM3 | PM | plasma | 408 | 3.02 |
| PM3 | PM | NKT cells | 1,001 | 7.42 |
| PM4 | PM | CD4 T | 1,625 | 19.50 |
| PM4 | PM | CD8 T | 1,016 | 12.19 |
| PM4 | PM | myeloid | 1,356 | 16.27 |
| PM4 | PM | granulocyte | 4,169 | 50.02 |
| PM4 | PM | B | 36 | 0.43 |
| PM4 | PM | plasma | 0 | 0.00 |
| PM4 | PM | NKT cells | 132 | 1.58 |
| PM5 | PM | CD4 T | 4,497 | 50.57 |
| PM5 | PM | CD8 T | 3,010 | 33.85 |
| PM5 | PM | myeloid | 151 | 1.70 |
| PM5 | PM | granulocyte | 6 | 0.07 |
| PM5 | PM | B | 96 | 1.08 |
| PM5 | PM | plasma | 1 | 0.01 |
| PM5 | PM | NKT cells | 1,132 | 12.73 |

*B. TBM donors*

| **Sample label** | **Diagnosis** | **Cell type** | **Cell count** | **Proportion of annotated major immune cells (%)** |
| --- | --- | --- | --- | --- |
| TBM1 | TBM | CD4 T | 3,401 | 41.57 |
| TBM1 | TBM | CD8 T | 3,355 | 41.00 |
| TBM1 | TBM | myeloid | 196 | 2.40 |
| TBM1 | TBM | granulocyte | 0 | 0.00 |
| TBM1 | TBM | B | 103 | 1.26 |
| TBM1 | TBM | plasma | 119 | 1.45 |
| TBM1 | TBM | NKT cells | 1,008 | 12.32 |
| TBM2 | TBM | CD4 T | 6,865 | 63.53 |
| TBM2 | TBM | CD8 T | 1,990 | 18.42 |
| TBM2 | TBM | myeloid | 3 | 0.03 |
| TBM2 | TBM | granulocyte | 25 | 0.23 |
| TBM2 | TBM | B | 512 | 4.74 |
| TBM2 | TBM | plasma | 9 | 0.08 |
| TBM2 | TBM | NKT cells | 1,402 | 12.97 |
| TBM3 | TBM | CD4 T | 5,405 | 54.93 |
| TBM3 | TBM | CD8 T | 4,046 | 41.12 |
| TBM3 | TBM | myeloid | 9 | 0.09 |
| TBM3 | TBM | granulocyte | 0 | 0.00 |
| TBM3 | TBM | B | 114 | 1.16 |
| TBM3 | TBM | plasma | 5 | 0.05 |
| TBM3 | TBM | NKT cells | 260 | 2.64 |
| TBM4 | TBM | CD4 T | 7,331 | 68.28 |
| TBM4 | TBM | CD8 T | 2,230 | 20.77 |
| TBM4 | TBM | myeloid | 420 | 3.91 |
| TBM4 | TBM | granulocyte | 5 | 0.05 |
| TBM4 | TBM | B | 330 | 3.07 |
| TBM4 | TBM | plasma | 22 | 0.20 |
| TBM4 | TBM | NKT cells | 399 | 3.72 |
| TBM5 | TBM | CD4 T | 5,038 | 65.37 |
| TBM5 | TBM | CD8 T | 2,045 | 26.53 |
| TBM5 | TBM | myeloid | 20 | 0.26 |
| TBM5 | TBM | granulocyte | 5 | 0.06 |
| TBM5 | TBM | B | 193 | 2.50 |
| TBM5 | TBM | plasma | 7 | 0.09 |
| TBM5 | TBM | NKT cells | 399 | 5.18 |

Note. Counts and proportions are shown at the donor level. Proportions were calculated relative to annotated major immune cells within each donor. For donor-level proportion comparisons, major immune-cell populations not detected in a given donor were assigned a value of zero. This zero assignment was used only for donor-level composition analysis and should not be interpreted as definitive biological absence of the corresponding cell population. Cell types include CD4⁺ T cells, CD8⁺ T cells, myeloid cells, granulocytes, B cells, plasma cells, and NKT-like cells. CSF, cerebrospinal fluid; PM, purulent meningitis; TBM, tuberculous meningitis.
