# Supplementary material for: Single-cell transcriptomic profiling of cerebrospinal fluid reveals immune-cell heterogeneity and multicompartment MIF/CD74-related communication in tuberculous and purulent meningitis
Source: Front Cell Infect Microbiol. 2026 Aug 13;16:1867160. doi: 10.3389/fcimb.2026.1867160 (PMC13518202; doi:10.3389/fcimb.2026.1867160)
Supplement: Supplementary file 3 [file DataSheet1.zip › data/CD74_Purity_Validation/02_Key_Markers_VlnPlot.pdf]

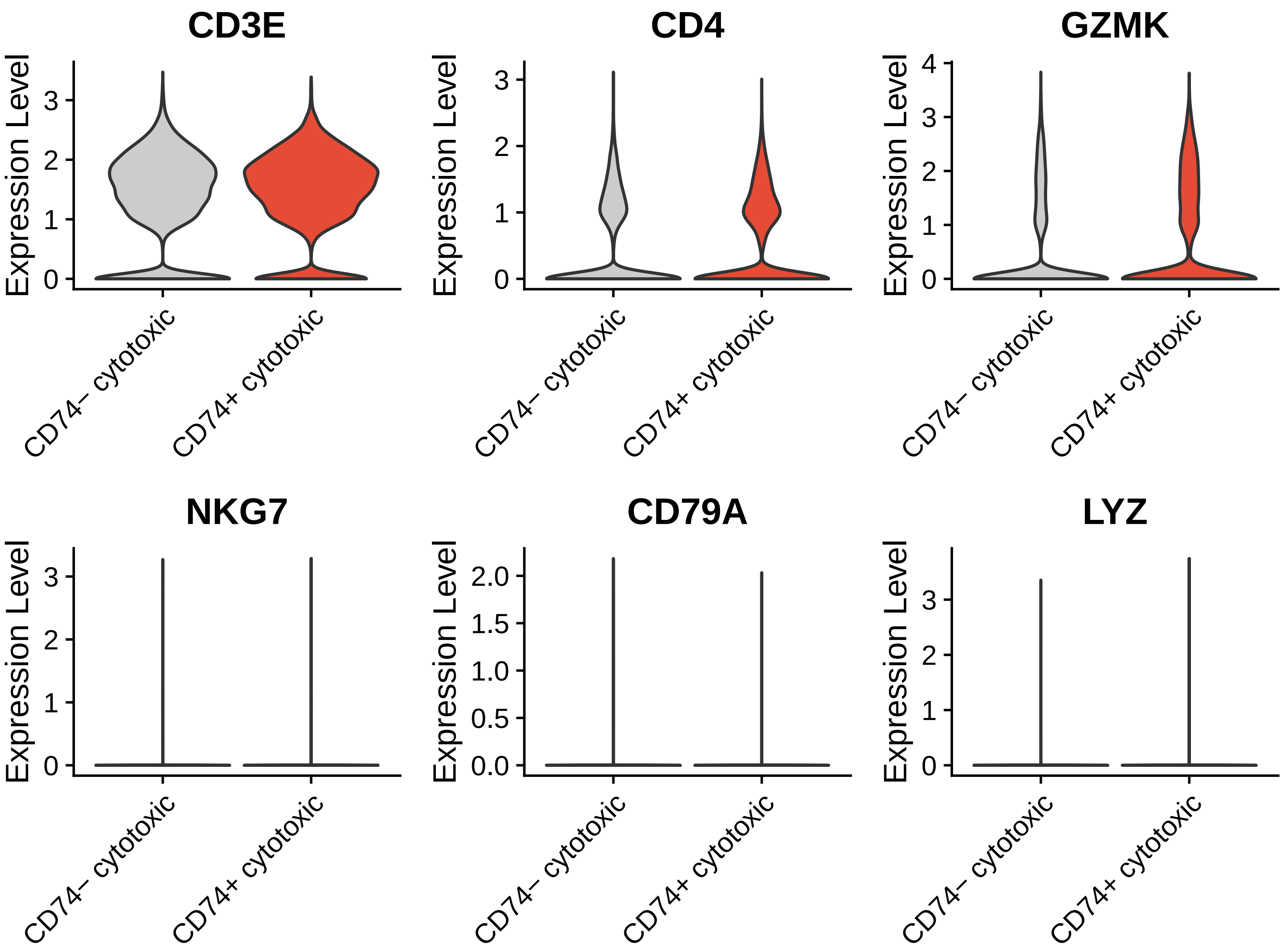
CD3E
CD4
GZMK
NKG7
CD79A
LYZ
Expression Level
0
1
2
3
4
0.0
0.5
1.0
1.5
2.0
CD74– cytotoxic
CD74+ cytotoxic
