# Supplementary material for: Single-cell transcriptomic profiling of cerebrospinal fluid reveals immune-cell heterogeneity and multicompartment MIF/CD74-related communication in tuberculous and purulent meningitis
Source: Front Cell Infect Microbiol. 2026 Aug 13;16:1867160. doi: 10.3389/fcimb.2026.1867160 (PMC13518202; doi:10.3389/fcimb.2026.1867160)
Supplement: Supplementary file 3 [file DataSheet1.zip › data/CD74_Purity_Validation/03_QC_Scores_Validation.pdf]

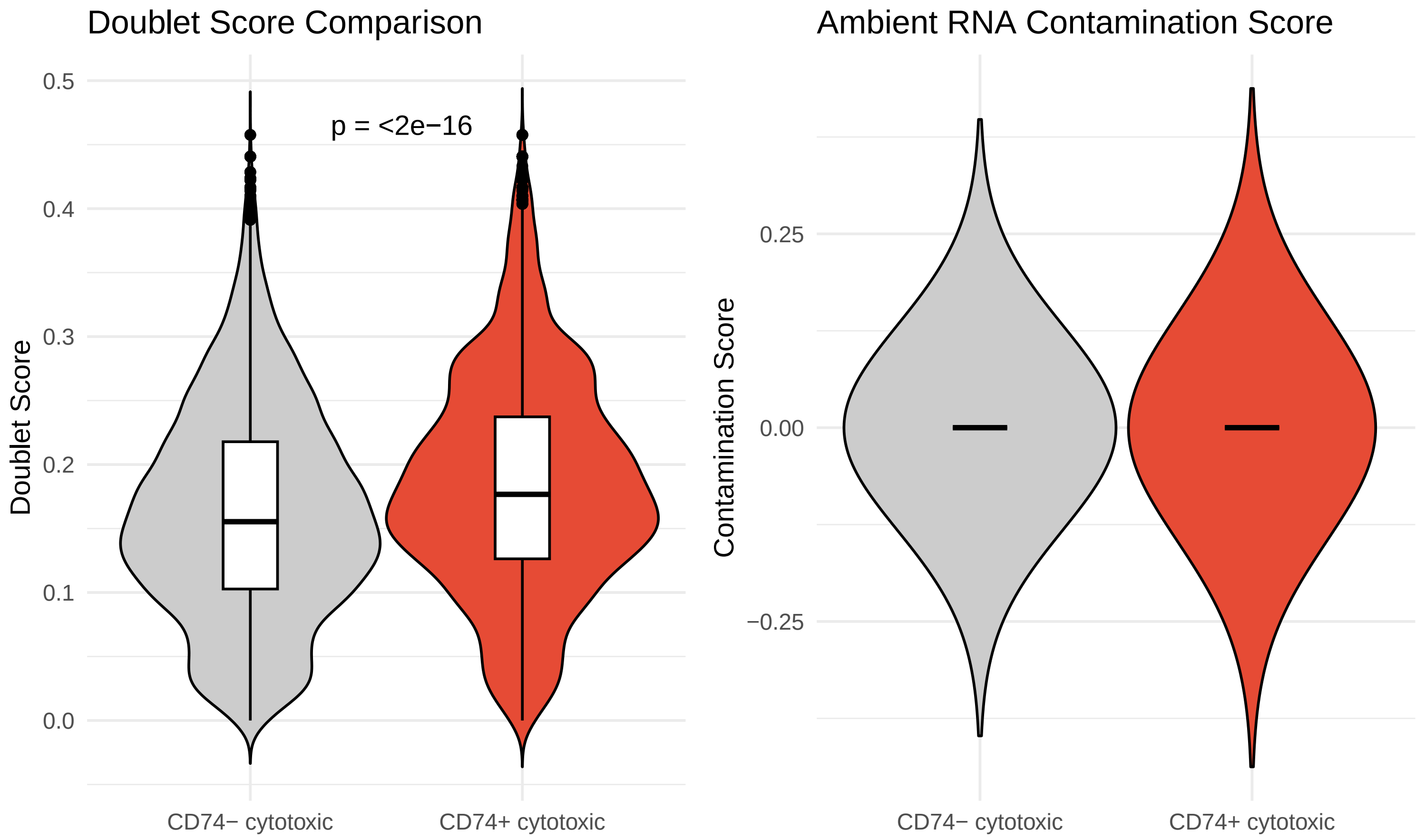

Doublet Score Comparison
p = <2e−16
Doublet Score
0.5
0.4
0.3
0.2
0.1
0.0
CD74− cytotoxic
CD74+ cytotoxic
Ambient RNA Contamination Score
Contamination Score
0.25
0.00
−0.25
CD74− cytotoxic
CD74+ cytotoxic
