# Supplementary material for: Single-cell transcriptomic profiling of cerebrospinal fluid reveals immune-cell heterogeneity and multicompartment MIF/CD74-related communication in tuberculous and purulent meningitis
Source: Front Cell Infect Microbiol. 2026 Aug 13;16:1867160. doi: 10.3389/fcimb.2026.1867160 (PMC13518202; doi:10.3389/fcimb.2026.1867160)
Supplement: Supplementary file 3 [file DataSheet1.zip › data/CD74_Purity_Validation/01_Marker_Validation_DotPlot.pdf]

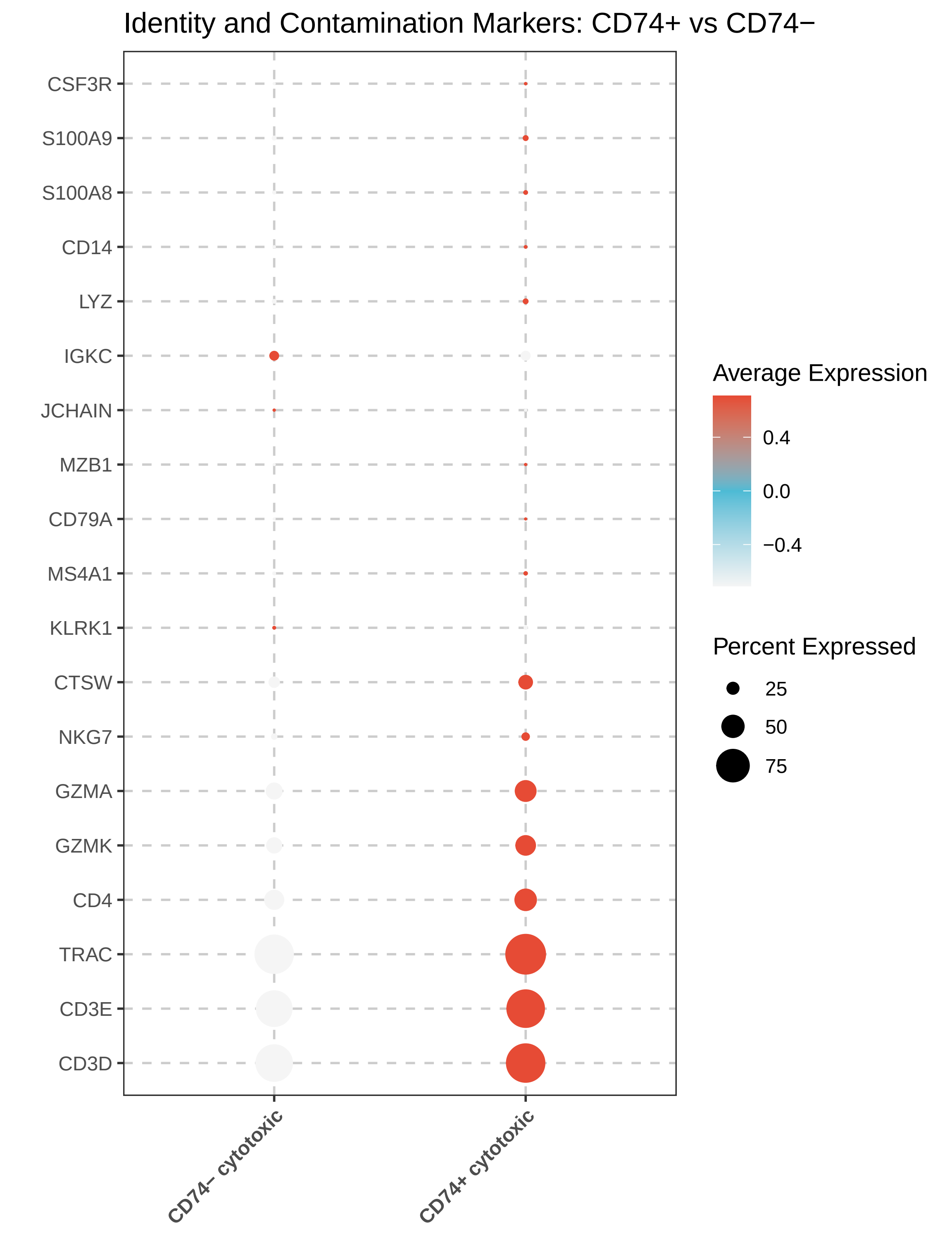
Identity and Contamination Markers: CD74+ vs CD74−
CSF3R
S100A9
S100A8
CD14
LYZ
IGKC
JCHAIN
MZB1
CD79A
MS4A1
KLRK1
CTSW
NKG7
GZMA
GZMK
CD4
TRAC
CD3E
CD3D
CD74− cytotoxic
CD74+ cytotoxic
Average Expression
0.4
0.0
−0.4
Percent Expressed
25
50
75
